# Benzyl Isothiocyanate potentiates p53 signaling and antitumor effects against breast cancer through activation of p53-LKB1 and p73-LKB1 axes

**DOI:** 10.1038/srep40070

**Published:** 2017-01-10

**Authors:** Bei Xie, Arumugam Nagalingam, Panjamurthy Kuppusamy, Nethaji Muniraj, Peter Langford, Balázs Győrffy, Neeraj K. Saxena, Dipali Sharma

**Affiliations:** 1 Department of Oncology, Johns Hopkins University School of Medicine and the Sidney Kimmel Comprehensive Cancer Center at Johns Hopkins, Baltimore MD 21231, USA; 2 Department of Medicine, University of Maryland School of Medicine, Baltimore MD 21201, USA; 3 MTA TTK Momentum Cancer Biomarker Research Group, H-1117 Budapest, Semmelweis University, 2nd Dept. of Pediatrics, H-1094 Budapest, Hungary

## Abstract

Functional reactivation of p53 pathway, although arduous, can potentially provide a broad-based strategy for cancer therapy owing to frequent p53 inactivation in human cancer. Using a phosphoprotein-screening array, we found that Benzyl Isothiocynate, (BITC) increases p53 phosphorylation in breast cancer cells and reveal an important role of ERK and PRAS40/MDM2 in BITC-mediated p53 activation. We show that BITC rescues and activates p53-signaling network and inhibits growth of p53-mutant cells. Mechanistically, BITC induces p73 expression in p53-mutant cells, disrupts the interaction of p73 and mutant-p53, thereby releasing p73 from sequestration and allowing it to be transcriptionally active. Furthermore, BITC-induced p53 and p73 axes converge on tumor-suppressor LKB1 which is transcriptionally upregulated by p53 and p73 in p53-wild-type and p53-mutant cells respectively; and in a feed-forward mechanism, LKB1 tethers with p53 and p73 to get recruited to p53-responsive promoters. Analyses of BITC-treated xenografts using LKB1-null cells corroborate *in vitro* mechanistic findings and establish LKB1 as the key node whereby BITC potentiates as well as rescues p53-pathway in p53-wild-type as well as p53-mutant cells. These data provide first *in vitro* and *in vivo* evidence of the integral role of previously unrecognized crosstalk between BITC, p53/LKB1 and p73/LKB1 axes in breast tumor growth-inhibition.

With approximately 50 to 55% human cancers exhibiting loss of wild-type p53 activity, tumor suppressor p53 is the most commonly silenced or mutated gene in cancer^1^,^2^. Acting as a transcription factor, p53, plays a critical role in suppressing growth, angiogenesis, invasion and migration as well as inducing apoptosis and growth-inhibition^3^,^4^, therefore cells deficient in normal p53-functioning can potentially undergo malignant transformation. Mice knockout for p53 are susceptible to spontaneous tumors^5^ and various studies utilizing *in vitro* model systems and mouse models have shown the functional relevance of reconstitution of p53-pathway to inhibit growth and progression of established tumors^6^,^7^. We aim to develop more-effective and non-toxic therapeutic strategies to achieve p53-activation using active constitutive agents in natural products owing to their cancer preventive as well as therapeutic potential. Bioactive components from plants have played an important role in the discovery and development of novel cancer preventive and therapeutic agents^8^,^9^. Dietary intake of cruciferous vegetables has been shown to have protective effects against the risk of various types of malignancies^10^,^11^. Anti-carcinogenic effect of cruciferous vegetables is due to chemicals with an isothiocyanate (ITC) functional group (N=C=S)^12^. Benzyl isothiocyanate (BITC) is an important ITC capable of inhibiting chemically-induced cancer in animal models^12^,^13^. BITC suppresses proliferation and induces apoptosis in multiple cancer-types^14^,^15^, including breast cancer^16^,^17^ but molecular understanding of BITC-mediated signaling-networks is still emerging. Investigating the potential of BITC to restore functionally-active tumor-suppressor p53 network and deciphering the key nodes of BITC-action in p53-activation will establish surrogate biomarkers for its efficacy and help in clinical development of this bioactive molecule, an issue we address by systematically elucidating the underlying mechanisms.

Modulation of phosphorylation-status of key proteins including kinases, oncogenes and tumor-suppressors is an important regulatory mechanism with functional consequences; therefore, in the present study we utilize phosphorylation-array to gain insight into the intricacies of BITC-induced signaling pathways and their impact on p53-signaling network. We discovered that BITC treatment alters phosphorylation status of extracellular-signal-regulated kinase (ERK), p53 and proline-rich Akt substrate of 40 kDa (PRAS40) in breast cancer cells. We designed this study to examine the role of tumor-suppressors p53 and p73 and the underlying molecular mechanisms how BITC-mediated activation of p53/p73 leads to growth-inhibition of breast cancer cells. Here, we provide strong evidence that BITC-induced p53 and p73 axes converge on tumor-suppressor LKB1, transcriptionally upregulating LKB1 in p53-wild-type and p53-mutant cells respectively. Our study uncovers that BITC concertedly modulates tumor-suppressors- p53, p73 and LKB1 and not only activates p53-signaling networks in p53-wild-type breast cancer but also functionally restores p53-signaling in p53-mutant breast cancer. Our study suggests that BITC could be a useful strategy to potentiate p53-signaling in p53-wild-type cells as well as rescue p53-signaling in p53-mutant cells hence offering a broad-based strategy that can be useful for multiple cancer types.

## Results

### Benzyl Isothiocyanate (BITC) treatment inhibits clonogenicity and anchorage-independent growth of breast cancer cells and breast tumor progression in athymic nude mice

**Figure 1:**
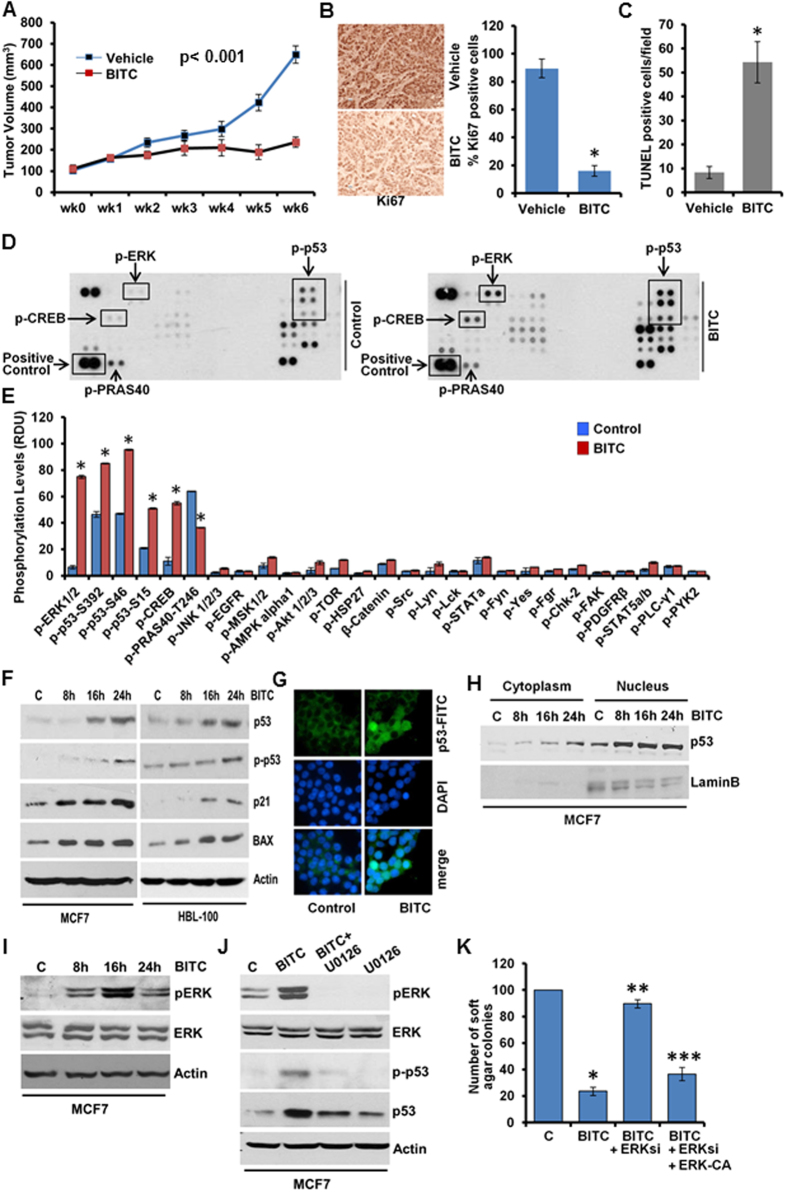
BITC inhibits breast tumor growth in nude mice. Human phospho-antibody array analyses reveal BITC-induced increased phosphorylation of p53 and ERK and BITC induces p53-phosphorylation in an ERK-dependent manner. (**A**) MCF7 cells derived tumors were developed in nude mice and treated with vehicle or BITC. Tumor growth was monitored by measuring the tumor volume for 6 weeks. (n = 8 mice/group); (P < 0.001). (**B**) Tumors from vehicle (V) and BITC-treated mice were subjected to immunohistochemical (IHC) analysis using Ki67 antibodies. Bar diagrams show quantitation of IHC-analysis. Columns, mean (n = 8); bar, SD. *significantly different (P < 0.005) compared with control. (**C**) TUNEL-positive cells in tumor sections were counted. Each bar represents the mean (n = 6–8). *P < 0.01, compared with controls. (**D,E**) MCF7 breast cancer cells were treated with 2.5 μM BITC and subjected to Human phospho-antibody array analyses. Relative levels of protein phosphorylation (normalized intensity for each antibody) were calculated for each untreated and treated sample. *P < 0.001, compared with controls. (**F**) Immunoblot analysis of phosphorylated-p53-Ser15 (p-p53), total p53, p21 and BAX in breast cancer cells treated with 2.5 μM BITC as indicated. (**G**) Breast cancer cells were treated with 2.5 μM BITC and subjected to immunofluorescence analysis of p53. (**H**) Breast cancer cells were treated with 2.5 μM BITC for various time-intervals as indicated followed by nuclear-cytoplasmic fractionation. Nuclear and cytoplasmic lysates were examined for p53. Lamin B and actin were included as controls. (**I**) Breast cancer cells were treated as in F, total lysates were immunoblotted for pERK and total ERK expression. (**J**) MCF7 cells were pretreated with 10 μM U0126 for 2 hours followed by treatment with 2.5 μM BITC. Total lysates were immunoblotted for pERK, total ERK, p-p53-Ser15 and total p53 expression. (K) MCF7 cells were transiently transfected with siERK-siRNAs for 48 h and subjected to colony-formation assay in the presence or absence of 2.5 μM BITC. Cells overexpressing ERK-CA are included as ‘gain-of-function’ controls. Histogram represents average number of colonies counted (in six micro-fields). *P < 0.001, compared with vehicle controls (**C**); **P < 0.005, compared with BITC-treated cells; ***P < 0.05, compared with BITC + ERKsi cells.

BITC treatment decreased cell-viability (Supplementary Figure 1A); clonogenicity and soft-agar colony-formation of breast cancer cells (Supplementary Fig. 1B,C). Mammosphere-forming capability of MCF7 and HBL-100 cells was also inhibited in response to BITC (Supplementary Figure 1D). BITC-mediated inhibition of cancer cell growth was associated with increased apoptotic cell death and induced PARP-cleavage (Supplementary Figure 1E,F,G). Next, we investigated the *in vivo* physiological relevance of our *in vitro* findings by evaluating whether oral-administration of BITC inhibits breast carcinoma in athymic nude mice. Growth of MCF7-xenografts was significantly inhibited in BITC-treated experimental group in comparison to the control group (Fig. 1A). Tumors from BITC-treated mice exhibited significantly lower Ki-67 (Fig. 1B), decreased expression of survivin and XIAP, members of inhibitor-of-apoptosis protein (IAP) family (Supplementary Figure 2A,B) and increased number of TUNEL-positive apoptotic cells compared with vehicle-control group (Fig. 1C). Collectively, these results show that BITC treatment results in suppression of tumor growth, inhibition of cellular proliferation and increased apoptosis in the breast tumors.

### Phosphokinase array analysis reveals modulation of distinct signaling mediators in response to BITC in breast cancer cells

Protein phosphorylation is fundamentally important to multiple aspects of signal-transduction pathways and cellular functions. We interrogated 46 specific Ser/Thr/Tyr phosphorylation sites of 38 selected proteins using phosphoprotein-arrays to identify cellular-signaling networks involved in BITC-mediated inhibition of breast carcinogenesis. Phosphorylation level of p53 was significantly increased in response to BITC treatment (Fig. 1D,E). In addition, BITC also increased phosphorylation of extracellular signal-regulated kinase (ERK) and cAMP-response-element-binding protein (CREB). Interestingly, decreased phosphorylation of proline-rich-Akt-substrate of 40 kDa (PRAS40) was observed upon BITC treatment (Fig. 1D,E). Tumor-suppressor p53^3^ is regulated at the posttranslational level by phosphorylation events that stablize and activate p53^18^. p53-phosphorylation increased in a tempral manner in MCF7 and HBL-100 cells upon BITC treatment (Fig. 1F) corroborating phosphokinase-array results. BITC-mediated p53-phosphorylation was accompanied with p53-stablization and activation as evident by increased p53 protein levels as well as elevated expression of p53-responsive genes-p21 and BAX (Fig. 1F). BITC did not affect p53 at the transcription level (Supplementary Figure 3). Nuclear localization of p53 is of major functional significance for activating or repressing p53-responsive transcriptional programs as well as preventing cytoplasmic proteolytic degradation 18. Immunofluoresecnce-analysis and immunoblotting of nuclear/cytoplasmic lysates of BITC-treated cells showed increased nuclear localization of p53 upon BITC treatment (Fig. 1G and H). We further explored the molecular mechanisms whereby BITC activates p53 and the biological significance of p53-activation in BITC-mediated inhibition of breast carcinogenesis.

### BITC treatment leads to stabilization and activation of p53 via extracellular signal-regulated kinase (ERK) and PRAS40 in breast cancer cells

**Figure 2:**
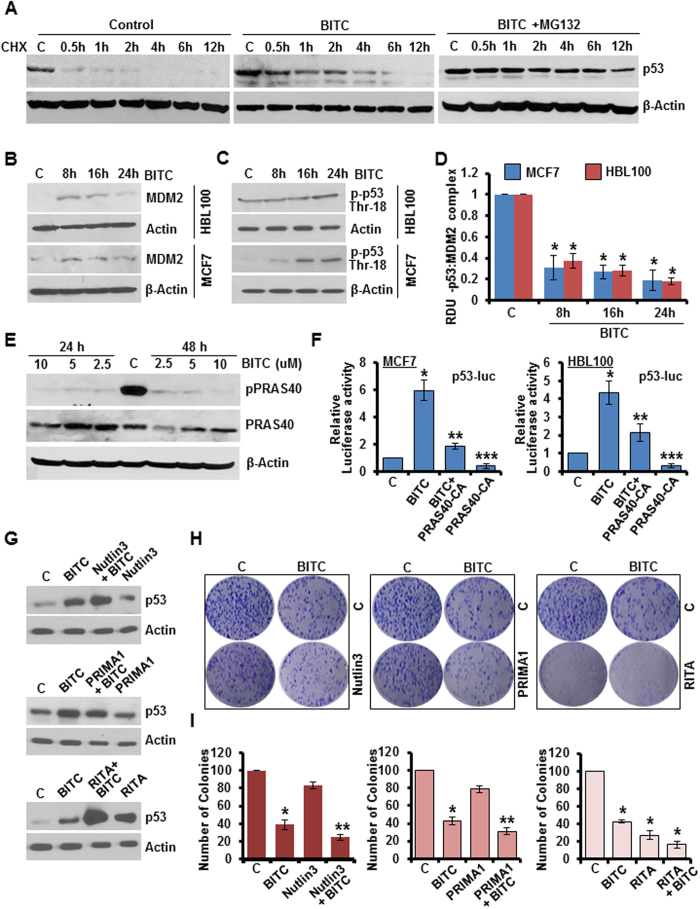
BITC induces stabilization of p53 via PRAS40 modulation. BITC is a potent activator of p53. **(A)** MCF7 breast cancer cells were treated with 20 μg/ml cycloheximide (CHX) in the presence of 2.5 μM BITC or 2.5 μM BITC and 20 μM MG132 and lysed at various time points as indicated. Total protein lysates were immunoblotted for p53 expression. Actin was used as control. **(B,C**) MCF7 and HBL100 cells were treated with 2.5 μM BITC as indicated and total protein lysates were immunoblotted for MDM2 and phospho-p53-Thr18 expression. **(D)** MCF7 and HBL100 cells were treated with 2.5 μM BITC, whole cell lysates were immunoprecipitated using MDM2 antibodies and purified immunoprecipitates were examined for p53 expression. IgG was used as control. Bar diagram shows quantitation of western blot signals from multiple independent experiments. **(E)** MCF7 cells were treated with various concentrations of BITC as indicated for 24 and 48 hours, total protein lysates were immunoblotted for phospho-PRAS40 and total PRAS40 expression. β-Actin was used as control. **(F)** MCF7 and HBL100 were transfected with p53-luc and/or constitutively-active PRAS40 (PRAS40-CA) and treated with 2.5 μM BITC as indicated followed by luciferase assay. **(G)** MCF7 cells were treated with 2.5 μM BITC, 5 μM Nutlin3, 25 μM PRIMA1, 0.05 μM RITA alone or in combination as indicated, cell lysates were examined for p53 expression. **(H,I)** MCF7 cells were treated as in G and subjected to clonogenicity. Bar-diagram shows percentage of number of colonies. *P < 0.001, compared with controls; **P < 0.005, compared with Nutlin3 or PRIMA1 alone; denoted with the letter “C”.

ERK-activation typically represents a major survival signaling pathway involved in promotion of cancer cell survival and inhibition of apoptosis, however, a pro-apoptotic role of ERK signaling has also been shown^19^. Phosphoprotein-array analysis showed elevated ERK-phosphorylation in breast cancer cells treated with BITC (Fig. 1D,E), a finding confirmed in immunoblot analysis (Fig. 1I). We queried the significance of ERK in BITC-mediated p53-phosphorylation. Immunoblot analysis for phosphorylated p53 levels showed that inhibition of ERK-phosphorylation with U0126 indeed inhibited BITC-induced p53-phosphorylation as well as total p53 levels (Fig. 1J). Exhibiting functional involvement, ERK-silencing using ERK-siRNA abrogated BITC-mediated inhibition of clonogenicity and soft-agar colony formation and also impeded BITC-induced apoptosis, importantly, reintroduction of constitutively-active-ERK (ERK-CA) in ERK-silenced cells re-sensitized them to BITC (Fig. 1K, Supplementary Figure 4A,B). Due to its role as a critical mediator of cell fate, p53 is subjected to multiple layers of regulation including transcriptional, translational and post-translational mechanisms. We sought to examine whether BITC controls p53 through the regulation of protein-stability. In the presence of translation-inibitor cycloheximide, BITC increased p53-protein half-life approximately eight-fold compared to vehicle-control (Fig. 2A). Cotreatment with the proteasome inhibitor MG132 further potentiated BITC-mediated p53-stabilization signifying the involvement of proteasome-mediated degradation of p53 in breast cancer cells (Fig. 2A). E3 ubiquitin-protein ligase Mouse double minute 2 homolog (MDM2), a p53-inducible gene, is known to negatively regulate p53 and maintain p53 at a low levels^20^. This negative regulation can be abrogated by p53-phosphorylation at Thr18 which blocks p53-MDM2 binding leading to an increase in p53 stability^21^. Treatment with BITC increased MDM2 expression in a temporal manner in HBL100 with maximum increase observed at 8 hours post-treatment interval. MCF7 cells also exhibited an increase in MDM2 expression in response to BITC treatment in a time-dependent manner (Fig. 2B). HBL100 cells exhibited an increase in p53-Thr18 phosphorylation upon 24 hours BITC treatment while the shorter duration of treatment remained ineffective. MCF7 cells showed increased p53-Thr18 phosphorylation upon 16 and 24 hours BITC treatment (Fig. 2C). BITC treatment prohibited MDM2-p53 binding/co-immunoprecipitation (Fig. 2D). Proline-rich Akt substrate of 40 kDa (PRAS40) is a major target of both Akt and mTORC1 and interestingly, is associated with promotion of cell survival and tumorigenesis unlike other inhibitors of mTORC1^22^. Recently, PRAS40 was found to negatively regulate p53 stability and this biological function of PRAS40 required phosphorylation at T246^23^. Phosphoprotein array analysis showed decreased phosphorylation of PRAS40 at T246 in breast cancer cells treated with BITC (Fig. 1D,E). Further exploring the involvement of PRAS40, we found that BITC-treatment indeed decreased phosphorylation of PRAS40-T246 (Fig. 2E) and overexpression of constitutively-active PRAS40 (PRAS40-CA) led to inhibition of BITC-induced p53 transactivation activity (Fig. 2F), and also abrogated BITC-mediated inhibition of clonogenicity and soft-agar colony formation (Supplementary Figure 5A,B). Collectively, these evidences support the notion that ERK, MDM2 and PRAS40 are functionally involved in BITC-induced p53-phosphorylation and stabilization and consequently participate in mediating biological effects of BITC.

### BITC is a potent inducer of p53 and silencing of p53 abrogates BITC-mediated growth-inhibition in p53-wild-type state

**Figure 3:**
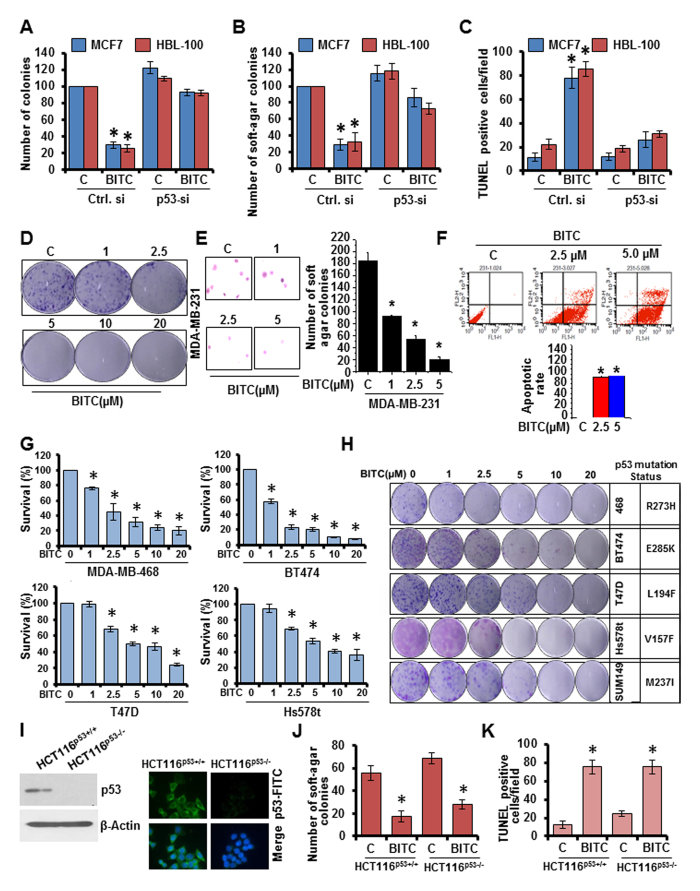
p53 plays an important role in mediating BITC-induced inhibition of growth in p53-wild-type breast cancer cells. BITC also inhibits growth and induces apoptosis in p53-mutant breast cancer cells and p53-null cells. (**A,B,C**) MCF7 and HBL100 cells were transiently transfected with p53-siRNA and control-si for 48 h and subjected to clonogenicity (**A**), soft-agar colony formation (**B**), and TUNEL staining in the presence of vehicle (**C**) or 2.5 μM BITC as indicated. *P < 0.001, compared with vehicle controls. (**D**) MDA-MB-231 cells were treated with various concentration of BITC and subjected to clonogenicity assay. (**E**) Soft-agar colony-formation of MDA-MB-231 cells treated with BITC for three weeks. Histogram represents average number of colonies counted (in six micro-fields). *P < 0.001, compared with controls. Vehicle-treated cells, denoted with the letter “C”. (**F**) MDA-MB-231 cells were treated with 2.5 μM BITC and subjected to Annexin V/PI staining. *p < 0.01, compared with controls. (**G**) MDA-MB-468, BT474, T47D and Hs578t cells were treated with various concentration of BITC as indicated and subjected to XTT assay. *P < 0.01, compared with controls. Vehicle-treated cells are denoted with the letter “C”. (**H**) p53 mutant breast cancer cells were treated with various concentration of BITC as indicated and subjected to clonogenicity assay. (**I**) Total protein was isolated from HCT116-p53 ^(+/+)^ and HCT116-p53 ^(−/−)^ cells and immunoblotted for p53 expression. HCT116-p53 ^(+/+)^ and HCT116-p53 ^(−/−)^ cells were also subjected to immunofluorescence analysis for p53 expression. (J) HCT116-p53 ^(+/+)^ and HCT116-p53 ^(−/−)^ cells were treated with 2.5 μM BITC and subjected to soft-agar colony formation assay. *p < 0.001, compared with controls. Vehicle-treated cells are denoted with C. (K) HCT116-p53 ^(+/+)^ and HCT116-p53 ^(−/−)^ cells were treated with 2.5 μM BITC and subjected to TUNEL assay. *p < 0.005, compared with controls. Vehicle-treated cells are denoted with C.

We compared the efficacy of BITC to induce p53 with established small-molecule p53-inducers (RITA, Nutlin3 and PRIMA1). BITC treatment resulted in greater induction of p53 expression in comparison to Nutlin3 and PRIMA1 while RITA-alone was found to be more effective than BITC. Combination treatment with BITC enhanced Nutlin3, PRIMA1 and RITA induced p53 expression (Fig. 2G) and resulted in significantly higher inhibition of clonogenicity and soft-agar colony formation (Fig. 2H,I). These results show that BITC is a potent inducer of p53 which can act as an effective bioactive alternative to RITA, Nutlin3 and PRIMA1 as well as enhance their effect when used in combination. Our results show that BITC tightly regulates p53 activation and accumulation at different levels indicating that p53 may serve as a key node in BITC’s anti-cancer function. Indeed, showing functional importance of p53, p53-silencing in p53-wild-type breast cancer cells (MCF7 and HBL-100), rendered them unresponsive to the inhibitory effects of BITC. BITC treatment inhibited clonogenicity, soft-agar colony formation and increased apoptotic death in control-si-transfected cells whereas p53-si-transfected cells remained unresponsive (Fig. 3A,B,C). Silencing of p53 in MCF7 cells did not induce p73 expression (Supplementary Figure 5C). These results show that anti-oncogenic effects of BITC in p53-wild-type cells are mediated via p53 activation.

### BITC is also capable of inhibiting growth in p53-mutant and p53-null state

Owing to the integral role of p53 in BITC-mediated growth-inhibition, we postulated that BITC would not be able to alter growth of p53-mutant breast cancer cells. We tested our hypothesis by treating p53-mutant MDA-MB-231 cells with BITC followed by functional assays. Contrary to our supposition, treatment with 2.5 μM BITC inhibited clonogenicity, soft-agar colony formation and mammosphere-formation of MDA-MB-231 cells (Fig. 3D,E, Supplementary Figure 6A) while known p53-inducers, RITA and PRIMA1 could not inhibit growth of p53-mutant cells (Supplementary Figure 6B). BITC also induced apoptosis in MDA-MB-231 cells (Fig. 3F). To further test that p53-independent actions of BITC are not limited to MDA-MB-231 cells, we treated multiple p53-mutant breast cancer cells with BITC and found that BITC could inhibit growth and clonogenicity of MDA-MB-468, BT474, T47D and Hs578t cells (Fig. 3G,H). Further, HCT116 p53^−/−^ and HCT116 p53^+/+^ cells (Fig. 3I) were treated with 2.5 μM BITC. BITC treatment inhibited soft-agar colony formation and induced apoptotic induction in both HCT116 p53^+/+^ and HCT116 p53^−/−^ cells (Fig. 3J,K). BAX and PUMA are important nodes of p53-network known to mediate its tumor-suppressor function^3^. We found that intact p53-signaling network is required for BITC-function as HCT116 PUMA^+/+^ and HCT116 BAX^+/+^ cells exhibited BITC-induced inhibition of clonogenicity while no significant inhibition was observed in HCT116 PUMA^−/−^ and HCT116 BAX^−/−^ cells (Supplementary Figure 7 and 8). Collectively, these results show that although p53 plays an important role in mediating BITC-induced growth-inhibition in p53-wild-type cells, BITC can also function in p53-independent manner suggesting an alternative signaling pathway and also show the requirement of intact p53-signaling network.

### Involvement of p73 in BITC-mediated p53-pathway-restoration and growth-inhibition of p53-mutant breast cancer cells

**Figure 4:**
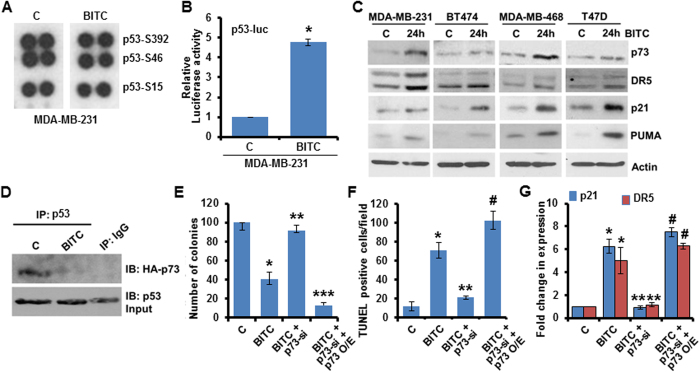
BITC doesn’t impact p53 expression but activates p53-signaling network in p53-mutant cells and p73 is functionally important for BITC. (**A**) MDA-MB-231 cells were treated with 2.5 μM BITC and subjected to Human phospho-antibody array analyses. Relative levels of protein phosphorylation (normalized intensity for each antibody) were calculated for each untreated and treated sample. Signals for p53 are shown. (**B**) MDA-MB-231 cells were transfected with p53-luc, treated with 2.5 μM BITC and subjected to luciferase assay. *P < 0.001, compared with vehicle-treated controls. (**C**) Total protein lysates from MDA-MB-231, BT474, MDA-MB-468 and T47D cells treated with 2.5 μM BITC were immunoblotted for p73, DR5, p21 and PUMA expression. β-actin was used as control. (**D**) MDA-MB-231 cells were transfected with HA-p73-full-length plasmid, treated with 2.5 μM BITC, whole cell lysates were immunoprecipitated using p53 antibodies and purified immunoprecipitates were examined for p73 expression. IgG was used as control. Vehicle-treated cells are denoted with the letter “C”. (**E**) MDA-MB-231 cells were transiently transfected with p73-siRNAs for 48 h and subjected to colony-formation assay in the presence or absence of 2.5 μM BITC. Cells overexpressing p73-full length are included as ‘gain-of-function’ controls. Histogram represents average number of colonies counted (in six micro-fields). *P < 0.005, compared with vehicle controls (**C**); **P < 0.01, compared with BITC-treated cells; ***P < 0.05, compared with BITC + p73si cells. (**F**) MDA-MB-231 cells were treated as in E and subjected to TUNEL assay. *P < 0.01, compared with vehicle controls (**C**); **P < 0.05, compared with BITC-treated cells; ^#^P < 0.001, compared with BITC + p73si cells. (**G**) MDA-MB-231 cells were treated as in E, total RNA was isolated and subjected to real-time PCR analysis for the expression of p21 and DR5. *P < 0.001, compared with vehicle controls (**C**); **P < 0.01, compared with BITC-treated cells; ^#^P < 0.001, compared with BITC + p73si cells.

Phosphoprotein arrays analysis of MDA-MB-231 cells treated with BITC showed no alteration in p53-phosphorylation levels (Fig. 4A). Despite the fact that no change in p53 was observed in BITC-treated p53-mutant breast cancer cells, we observed that BITC-induced p53-like transcriptional activity resulting in upregulation of p53-target proteins. p53-mutant, MDA-MB-468, BT474, T47D and Hs478t cells exhibited increased p53-transactivation activity and expression of p53-target proteins -DR5, p21 and PUMA upon BITC treatment (Fig. 4B,C). These results suggested that BITC can restore p53-transcriptional response in a p53-independent manner. A member of p53 family, p73 is a transcription factor with high structural and functional homology with p53. P73 gets recruited to promoters of p53-target genes, regulates their transcriptional activation and affect cell-growth and cell-death pathways in a manner similar to p53^24^,^25^. Therefore, we hypothesized that BITC might induce p73 expression to activate p53 pathway signaling, leading to growth-inhibition of p53-mutant breast cancer cells. Indeed, BITC treatment induced p73 expression in p53-mutant breast cancer cells (Fig. 4C). Mutant-p53 interacts with p73 to form an inhibited complex with respect to the transactivation of target genes and abrogates p73 function^26^. We found that BITC can disrupt the interaction of mutant-p53/p73 complex in an immunoprecipitation assay (Fig. 4D). To further confirm that p73 plays a role in BITC-mediated p53-pathway activation, growth-inhibition and apoptosis-induction in p53-mutant breast cancer cells, we silenced p73 using siRNA or overexpressed p73 using HA-p73 overexpression construct (HA-p73 O/E) in MDA-MB-231 cells. When p73 was silenced in MDA-MB-231 cells, BITC was not able to potentiate the expression of p53-target genes (Fig. 4G). Also, BITC treatment did not inhibit growth and increase apoptosis in p73-silenced MDA-MB-231 cells (Fig. 4E,F). In contrast, p73-overexpression in MDA-MB-231 cells further enhanced BITC-mediated increased expression of p53-target genes (p21, DR5), and resulted in more effective growth-inhibition and apoptosis-induction in response to BITC (Fig. 4E,F,G). Together, these evidences indicate the involvement of p73-upregulation in the mechanism whereby BITC activates p53-pathway, inhibits growth and induces apoptosis in p53-mutant breast cancer cells.

### LKB1 is an important node in molecular mechanisms underlying BITC’s anti-cancer role

**Figure 5:**
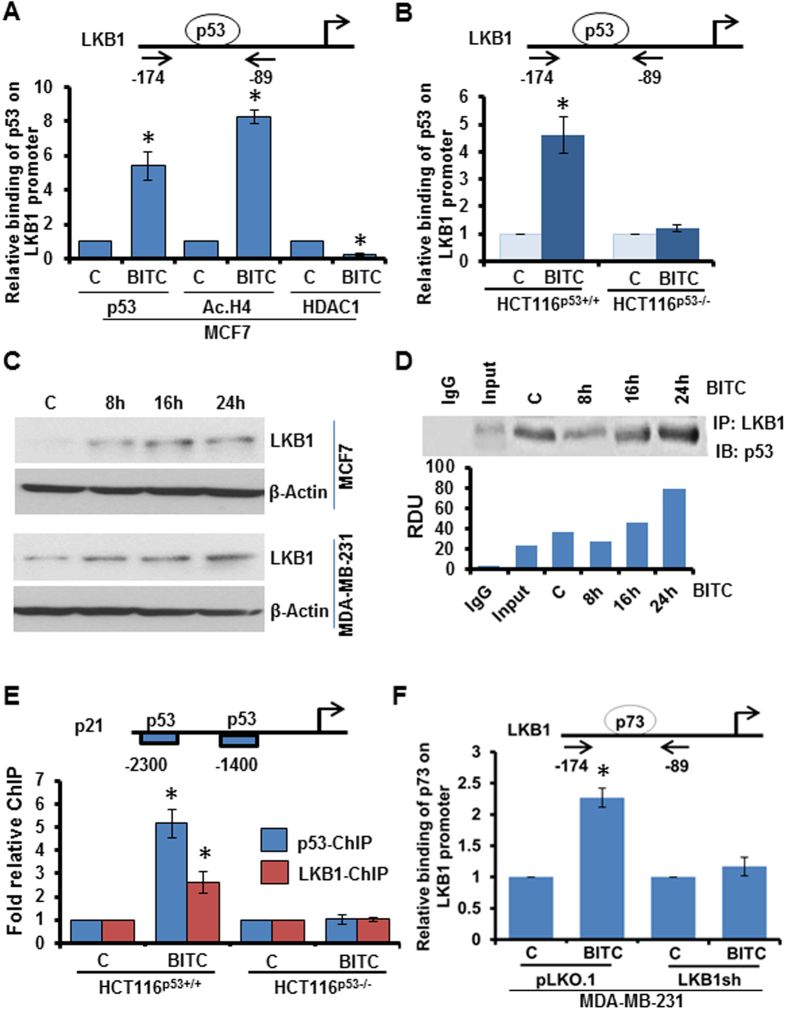
BITC induces functional interactions between p53, p73 and LKB1. **(A)** Soluble chromatin was prepared from MCF7 cells treated with 2.5 μM BITC as indicated and subjected to chromatin immunoprecipitation assay using p53, Acetylated-histone H4 (Ac H4) and HDAC1 antibodies. IgG antibody was included as control. The purified DNA was analyzed by real-time quantitative PCR using primers spanning the p53-binding sites at LKB1 promoter. *P < 0.001, compared with vehicle controls. **(B)** HCT116-p53 ^(+/+)^ and HCT116-p53 ^(−/−)^ cells treated with 2.5 μM BITC were subjected to chromatin immunoprecipitation assay using p53 antibody. IgG antibody was included as control. The purified DNA was analyzed by real-time quantitative PCR using primers spanning the p53-binding sites at LKB1 promoter. *P < 0.001, compared with vehicle controls. **(C)** MCF7 cells were treated with various concentrations of BITC as indicated for 24 and 48 hours, total protein lysates were immunoblotted for LKB1 expression. β-Actin was used as control. Bar diagram shows quantitation of western blot signals from multiple independent experiments. **(D)** MCF7 cells were treated with 2.5 μM BITC, whole cell lysates were immunoprecipitated using LKB1 antibodies and purified immunoprecipitates were examined for p53 expression. IgG was used as control. Bar diagram shows quantitation of western blot signals from multiple independent experiments. **(E)** HCT116-p53 ^(+/+)^ and HCT116-p53 ^(−/−)^ cells treated with 2.5 μM BITC were subjected to chromatin immunoprecipitation assay using p53 and LKB1 antibodies. IgG antibody was included as control. The purified DNA was analyzed by real-time quantitative PCR using primers spanning the p53-binding sites at p21 promoter. *P < 0.005, compared with vehicle controls. **(F)** MDA-MB-231 cells treated with 2.5 μM BITC were subjected to chromatin immunoprecipitation assay using p73 antibody. IgG antibody was included as control. The purified DNA was analyzed by real-time quantitative PCR using primers spanning the p53-binding sites at LKB1 promoter. *P < 0.001, compared with vehicle controls.

Liver kinase B1 (LKB1) is an important upstream-kinase and tumor-suppressor regulating several downstream pathways and known tumor-suppressors^27^. Analysis of LKB1 promoter revealed 4 potential binding sites for p53^28^. ChIP analyses showed that p53 gets recruited to −108 to −88 bp promoter region of LKB1 in breast cancer cells treated with BITC whereas untreated cells showed no p53 binding to LKB1 promoter. HCT116p53+/+ also showed p53 binding to LKB1 promoter. p53−/− cells were included as negative controls for ChIP assay. BITC treatment released HDAC1 from LKB1 promoter and significantly increased histone H4 acetylation indicating active chromatin conformation (Fig. 5A,B). Indeed, BITC treatment increased the expression of LKB1 (Fig. 5C). In addition, LKB1 has been shown to associate with p53 and participate in p53-mediated transactivation function^29^. Testing BITC-induced interaction of LKB1 and p53 in an immunoprecipitation assay, we found that BITC increased the association of p53 and LKB1 in breast cancer cells (Fig. 5D). We questioned whether LKB1 associates with p53 and get recruited to p53-responsive genes. Analysis of chromatin immunoprecipitates showed that LKB1 bound to p53-response elements of p53-target gene, p21, along with the recruitment of p53 in breast cancer cells treated with BITC (Fig. 5E). In an effort to better understand the molecular events involved in BITC-treated p53-mutant cells, we examined if p73 mimics p53 actions on LKB1 promoter. Interestingly, we found that BITC treatment induced the recruitment of p73 on p53-response element on LKB1 promoter in p53-mutant MDA-MB-231 cells (Fig. 5F). Also, p73 and LKB1 associated with p53-response elements on p21 promoter in p53-mutant breast cancer cells upon BITC treatment (Supplementary Figure 9). Taken together, these data show that p53 and p73 transcriptionally upregulate tumor-suppressor LKB1 in p53-wild-type and p53-mutant breast cancer cells respectively; and in a feed-forward mechanism, LKB1 tethers with p53 and p73 to get recruited to p53-responsive gene promoters.

### Silencing of LKB1 abrogates BITC-mediated growth-inhibition of breast cancer *in vitro* and *in vivo*

**Figure 6:**
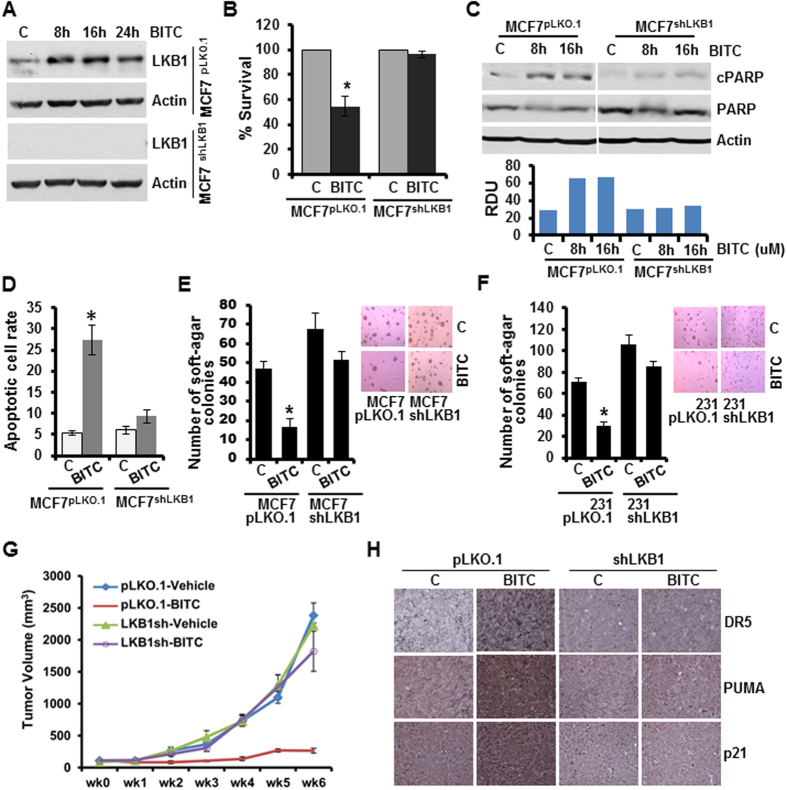
LKB1 is involved in BITC-mediated growth-inhibition and apoptotic-induction in breast cancer cells. **(A)** Total protein lysates of LKB1-depleted (LKB1^shRNA^) and vector control (pLKO.1) MCF7 cells were immunoblotted for the expression of LKB1. MCF7-LKB1^shRNA^ and MCF7- pLKO.1 cells were treated with 2.5 μM BITC and total protein lysates were examined for LKB1 expression in an immunoblot assay. β-actin was used as control. **(B)** MCF7-LKB1^shRNA^ and MCF7- pLKO.1 cells were treated with 2.5 μM BITC and subjected to XTT assay. *p < 0.01, compared with untreated controls. Vehicle-treated cells are denoted with the letter “C”. **(C)** Total protein lysates from MCF7-LKB1^shRNA^ and MCF7- pLKO.1 cells treated with 2.5 μM BITC for 8 and 16 hours were immunoblotted for the expression of cleaved PARP and total PARP. β-actin was used as a control. Bar diagram shows quantitation of western blot signals from multiple independent experiments. **(D)** MCF7-LKB1^shRNA^ and MCF7- pLKO.1 cells were treated with 2.5 μM BITC and subjected to Annexin V/PI staining. *p < 0.01, compared with untreated controls. **(E)** MCF7-LKB1^shRNA^ and MCF7- pLKO.1 cells were treated with 2.5 μM BITC and subjected to soft-agar colony formation assay. *p < 0.01, compared with untreated controls. **(F)** MDA-MB-231-LKB1^shRNA^ and MDA-MB-231-pLKO.1 cells were treated with 2.5 μM BITC and subjected to soft-agar colony formation assay. *p < 0.01, compared with untreated controls. (**G)** LKB1-depleted (LKB1^shRNA^) and vector control (pLKO.1) MDA-MB-231 cells derived tumors were developed in nude mice and treated with vehicle and BITC. Tumor growth was monitored by measuring the tumor volume for 6 weeks. (n = 8–10); (*p* < 0.001), pLKO.1+ BITC compared with LKB1^shRNA^ + BITC. **(H)** Tumors from LKB1^shRNA^+Vehicle, pLKO.1+Vehicle, LKB1^shRNA^ + BITC, and pLKO.1+ BITC groups were subjected to Immunohistochemical (IHC) analysis using p21, PUMA and DR5 antibodies.

**Figure 7:**
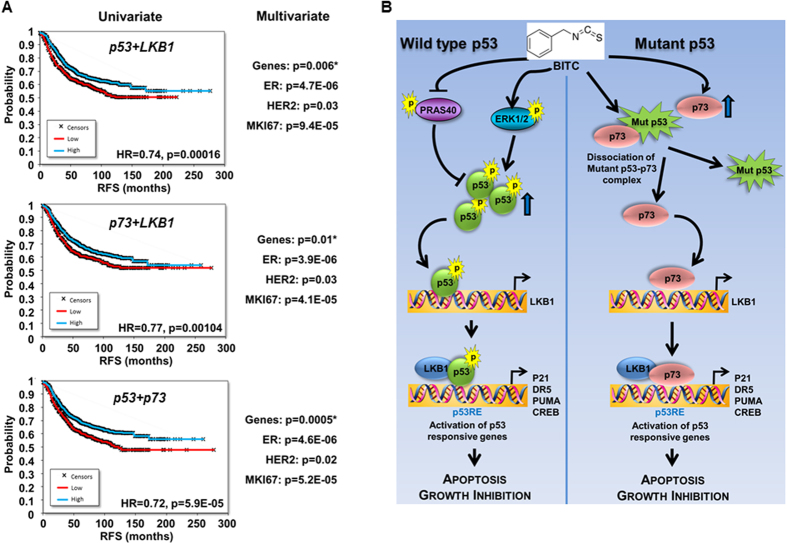
Survival analyses of p53, p73 and LKB1 and schematic representation of BITC-mediated crosstalk between p53, LKB1 and p73. **(A)** Survival analyses in 1,819 patients for TP53+LKB1, TP73+LKB1, and for TP53+TP73. Univariate analysis was performed using the combination of the two genes in patients with relapse-free survival data. Multivariate regression analysis involving estrogen receptor and HER2 receptor status and MKI67 expression. **(B)** BITC treatment inhibits PRAS40 and induces phosphorylation of ERK leading to increased phosphorylation and accumulation of p53. BITC also induces recruitment of p53 to p53-response element (p53RE) on LKB1 promoter leading to increased LKB1 expression which in turn forms a complex with p53 and gets recruited to p53RE on p53-responsive genes. In p53-mutant breast cancer cells, BITC increases p73 expression as well as abrogates the complex between mutant-p53 and p73 hence freeing p73 and stimulates p73 recruitment to p53RE on LKB1 promoter. LKB1 and p73 tether and get recruited to p53-responsive genes in the presence of BITC. BITC induces apoptosis and inhibits growth of p53-wild-type as well as p53-mutant breast cancer cells via mediating crosstalk between p53, LKB1 and p73.

To directly examine the role of LKB1 in BITC-mediated growth-inhibition of breast cancer cells, stable pools of breast cancer cells with LKB1 depletion were developed using LKB1^shRNA^ lentiviruses and puromycin selection. We analyzed pLKO.1 and LKB1^shRNA^ stable MCF7 and MDA-MB-231 cell pools for LKB1 protein expression, and found that LKB1 protein expression was significantly knocked-down in LKB1^shRNA^ cells as compared to pLKO.1 control cells. BITC treatment increased LKB1 expression as expected in MCF7^pLKO.1^ cells (Fig. 6A). BITC decreased growth of MCF7^pLKO.1^ cells whereas LKB1^shRNA^ cells remained unaffected by BITC treatment (Fig. 6B). Also, BITC increased PARP cleavage in pLKO.1 cells whereas no change in cleaved PARP was observed in LKB1^shRNA^ breast cancer cells (Fig. 6C). Increased apoptotic cell rate observed upon BITC treatment in pLKO.1 breast cancer cells was abrogated upon LKB1 silencing (Fig. 6D). BITC treatment efficiently inhibited soft-agar-colony formation of pLKO.1 breast cancer cells (MCF7 and MDA-MB-231) but not of LKB1^shRNA^ cells (Fig. 6E,F). Next, we investigated the *in vivo* physiological relevance of our *in vitro* findings by evaluating whether LKB1 is integral for the inhibitory effects of BITC on the development of breast carcinoma in nude mouse models. MDA-MB-231-pLKO.1 and MDA-MB-231-LKB1^shRNA^ were utilized in xenograft-athymic nude mice model. Breast tumor growth was significantly inhibited in MDA-MB-231-pLKO.1 (vector control) group upon BITC treatment whereas BITC was unable to inhibit tumor growth in MDA-MB-231-LKB1^shRNA^ group (Fig. 6G). Immunohistochemical analysis of LKB1^shRNA^ tumors (Vehicle and BITC groups) did not show any significant change in p21, DR5 and PUMA expression. Tumors from BITC-pLKO.1 group exhibited higher number of tumor cells showing increased expression of p21, DR5 and PUMA as compared to tumors from vehicle-treated group (Fig. 6H) providing physiological relevance to our *in vitro* findings. Moreover, a strong correlation exists between elevated p53, p73 and LKB1 and better prognosis for breast cancer patients. Univariate analysis was performed using the combination of the two genes in patients with relapse-free survival data. Multivariate Cox regression analysis was performed for each combination using ER status, HER2 status and MKI67 expression as a surrogate marker for proliferation. The correlation to survival retained significance in a Cox multivariate regression analysis involving estrogen receptor and HER2 receptor status and MKI67 expression (Fig. 7A). Collectively, the findings presented here suggest that BITC inhibits breast tumor progression and provide *in vitro* as well as *in vivo* evidence for the involvement of LKB1 as an important mediator, and uncover a novel mechanism of BITC action through p53 and p73 activation.

## Discussion

Recent renewed interest in non-toxic, non-endocrine agents to effectively activate p53-networks to prevent and inhibit breast carcinogenesis has made benzyl isothiocyanate (BITC) of potential interest and sparked a new interest in understanding the underlying molecular mechanisms. We recognize that elucidating the key nodes of BITC action can also help establish surrogate biomarkers, a task essential for the clinical development of this bioactive molecule. Our results show that BITC treatment effectively inhibits the growth of breast cancer cells *in vitro* and *in vivo* but our phosphoprotein array studies led us to novel discoveries that extend beyond the popular notion of inhibiting oncogenic signals to achieve tumor-inhibition. ERK is popularly known for its pro-proliferation role but this study illustrates that BITC despite effectively inhibiting breast tumor growth, increases ERK-phosphorylation. We report an interesting mechanism whereby BITC-induced ERK modulates phosphorylation of tumor-suppressor p53 and along with a concerted action of PRAS40/MDM2, results in p53 accumulation and activation. Furthermore, these studies show that BITC induces the expression of p73 in p53-mutant breast cancer cells, disrupts the p73:mutant-p53 sequestration complex thereby releasing p73 to drive p53-network leading to tumor inhibition. BITC-induced p53 and p73 axes congregate on tumor-suppressor LKB1 which associates with p53 and p73 to regulate p53-signaling network. We also identify BITC as a potent p53-activator whose effects mimic molecular and cell-biological outcomes of established-p53-activators. *In vivo* analyses of tumor xenografts provide further evidence of an integral role of p53-network. Based on these data, we provide a schematic-diagram depicting a series of events including a feed-forward interaction of ERK, involvement of MDM2/PRAS40/ERK in p53 upregulation, participation of p73 and LKB1 which is operative in BITC-induced p53-signaling networks-dependent growth-inhibition of breast cancer cells (Fig. 7). We present BITC as a non-toxic, effective bioactive strategy to orchestrate interactions of three tumor-suppressors.

Despite associated difficulties and failures, restoring tumor-suppressors and activating their signaling-networks in cancer cells still holds tremendous therapeutic and preventative potential. Tumor-suppressor p73 is a p53-family protein that not only shares remarkable homology in DNA sequence and protein structure with p53 but also regulates similar gene-networks by recruiting to p53-response elements within gene promoters^30^. Though functionally similar to p53, p73 is rarely deleted or mutated in human cancer^30^. Activation of p73 has been implicated in apoptosis induction in cancer cells^26^,^31^. Mutant-p53 binds to p73 to form an inhibited complex, consequently sequestering p73 and blocking transactivation of target genes and downstream biological functions^26^ hence disrupting interaction of mutant-p53 and p73 leading to p73 release is a promising strategy to restore p53-pathway in cancer therapy. There has been a lot of interest in examining p73 as a bona fide attractive target in p53-mutant cancer cells. Our studies provide a new mechanism by which BITC can achieve effective breast tumor growth-inhibition in p53-mutant breast cancer by activating p73 and disrupting mutant-p53:p73 inhibited complex. In fact, in comparison to other p73-targeting agents such as SIMPs^31^ and RETRA^32^, known to target p73 by disrupting mutant-p53:p73 inhibited complex hence effective only in p53-mutant cancer cells, BITC is more beneficial, as BITC not only disrupts mutant-p53:p73 association but also effectively upregulates p73 expression.

Activation of p53 and rescue of deficient p53 function has been recognized as an effective way to therapeutically target cancer cells but this notion is marred by inherent complexities of the mechanisms leading to p53-loss as well as by the toxicities associated with therapeutic agents. Over the few years, several small molecules have been examined to target protein-protein interactions and protein-folding pathways involved in p53 regulation such as ABT-373, RITA, MI-219 and nutlin to block protein-protein interactions^33^,^34^ and PRIMA-1 and CP-31398^35^,^36^ to target protein folding. Nutlin analog RG7112 resulted in stable disease in 14 out of 20 liposarcoma patients and a partial response in one patient but all patients experienced adverse effects such as neutropenia and thrombocytopenia^37^. An ideal strategy to activate/rescue functional p53-network is the one that can not only potentiate wild-type p53 but also overcome multiple different mechanisms underlying p53-loss-of-function. Here, we show that BITC is more effective than other known p53 activators, PRIMA-1^MET^ and Nutlin3, in activation of p53-network in breast cancer cells hence, appears to be a good candidate for further development as an effective p53-targeting agent. It is interesting to note that in recent years, many phase 1 and 2 single agent and combination clinical trials have been conducted to examine the efficacy and safety of p53 activators^38^,^39^. Studies with these p53-targeting agents have been encouraging but no phase 3 study has yet been completed leading to approval for clinical use in breast cancer suggesting the need for novel agents.

Also, we show that ERK activation which is generally considered a survival-signaling pathway is important for BITC action in breast cancer cells. Kinetics and duration of ERK activation are important for ERK-mediated induction of apoptotic pathways^19^ as an early transient activation of ERK inhibits cell death^40^ while prolonged late ERK activation has been associated with the proapoptotic effect of ERK^19^. BITC induces a sustained ERK activation in breast cancer cells leading to p53-phosphorylation aiding its stabilization and accumulation. In our study, inhibition of ERK with both small molecule inhibitors and siRNA -mediated gene silencing abolishes BITC-mediated p53-induction and growth-inhibition of breast cancer cells. Moreover, we report a novel finding that tumor-suppressor LKB1 is integral for BITC action in breast cancer cells. LKB1 serves as a major hub regulating several downstream pathways and known tumor-suppressors owing to its dual role as a tumor-suppressor and an upstream master kinase^27^. Low LKB1 protein levels correlate with poor prognosis in breast carcinoma^41^ and LKB1 activation has been associated with better clinical outcomes. In conclusion, our studies reveal the involvement of a previously unrecognized functional crosstalk between BITC and p53/LKB1 and p73/LKB1 where LKB1 plays an integral role in mediating BITC’s anti-tumor effects *in vivo*. BITC could serve as a promising lead compound for the development of anti-cancer drugs that can potentiate as well as rescue p53-networks in breast cancer cells harboring p53-wild-type, p53-null and p53-hot-spot mutations.

## Materials and Methods

### Cell culture and reagents

MCF7, HBL-100, MDA-MB-231, MDA-MB-468, BT474, T47D, and Hs578t human breast cancer cell lines were obtained from the American Type Culture Collection (ATCC, Manassas, VA). HCT116^p53−/−^, HCT116^p53+/+^, HCT116^PUMA+/+^, HCT116^PUMA−/−^, HCT116^BAX+/−^ and HCT116^BAX−/−^ cells were kindly provided by Dr. Bert Vogelstein (Johns Hopkins University, MD). Benzyl Isothiocyanate (BITC) was procured from LKT Laboratories (St. Paul, MN, USA). Antibodies for p53, phospho-p53-ser15, phospho-p53-ser18, p21, BAX, Lamin B, phospho-ERK, ERK, phospho-PRAS40, PRAS40, MDM2, PUMA, cleaved PARP, PARP, DR5, LKB1 and p73 were purchased from Cell Signaling Technology (Danvers, MA). β-Actin antibody was purchased from Sigma-Aldrich (St. Louis, MO).

### Clonogenicity, anchorage-independent growth, cell-viability, mammosphere and apoptosis assays

To perform *clonogenicity assay*^42^, breast cancer cells were treated with BITC as indicated for 10-days; colonies were counted. *Anchorage-independent growth* of breast cancer cells in the presence of BITC was assayed by colony formation in soft agar^43^. *Cell viability assay* was performed using a commercially available XTT assay kit (Roche Applied Science, Indianapolis, IN). *Mammosphere* assays were performed as previously described^44^ and spheres (>50 μm) were counted. For *apoptosis* analysis, cells were stained with Annexin-V and propidium iodide (PI) followed by fluorescence-activated cell sorting (FACS) analysis.

### Breast tumorigenesis assay

MCF7, MDA-MB-231, MDA-MB-231-pLKO.1, and MDA-MB-231-LKB1^shRNA^ xenografts were generated in athymic nude mice as previously described^43^, grouped in 2 experimental groups (8 mice/group) and treated with oral gavage of either vehicle (20% intralipid) or vehicle containing 9 μmoles BITC (ChromaDex Inc., Irvine, CA)/kg body weight 5 days/week for 6 weeks^45^. Tumors were collected after 6 weeks of treatment; measured, weighed, and subjected to further analysis. For IHC, at least four random, nonoverlapping representative images from each tumor section from eight tumors of each group were captured using ImagePro software for quantitation of Ki-67, p21, DR5 and PUMA expression. All animal studies were in accordance with the animal protocol approved by Johns Hopkins University Animal Care Use Committee.

### Phospho-Antibody Array Analysis

Breast cancer cells were treated with BITC and the phospho-antibody array analysis was performed using the Proteome Profiler Human Phospho-Kinase Array Kit ARY003 from R&D Systems according to the manufacturer’s instructions. Array images were analyzed using the GeneTools image analysis software (Syngene).

### Subcellular fractions, Immunoprecipitation and Immunoblotting

Cellular cytosolic and nuclear fractions were prepared following previously published protocol^46^. Whole cell lysate was prepared using modified-RIPA buffer^47^ followed by immunoblotting. For immunoprecipitation, whole cell lysates were incubated with specific antibodies and protein-antibody complexes were immune precipitated with protein A/G agarose beads. Purified complexes were analyzed using immunoblot analysis.

### Immunofluorescence and confocal imaging

Fixed and immunofluorescently stained cells were imaged using a Zeiss LSM510 Meta (Zeiss) laser scanning confocal system configured to a Zeiss Axioplan 2 upright microscope^48^. All experiments were performed multiple times using independent biological replicates.

### LKB1 stable knockdown using Lentiviral short hairpin RNA

Five pre-made lentiviral LKB1 short hairpin RNA (shRNA) constructs and a negative control construct created in the same vector system (pLKO.1) were purchased from Open Biosystems (Huntville, AL). Constructs were used for transient transfection using Fugene or Lipofectamine. Paired LKB1 stable knockdown cells (MCF7) were generated following our previously published protocol^49^.

### Chromatin immunoprecipitation (ChIP), RNA-isolation and RT-PCR

ChIP analyses were performed using our published procedure^50^. For RNA isolation^51^ and RT-PCR, total cellular RNA was extracted using the TRIzol Reagent (Life Technologies, Inc., Rockville, MD). RT-PCR was performed using specific sense and antisense PCR primers.

### Gene chip database construction

To validate the correlation between clinical outcomes related to genes influenced by BITC, we performed a meta-analysis of publicly available datasets. A gene expression database comprising microarray data with clinical annotation for breast cancer patients was setup up as described previously^52^. Kaplan-Meier survival plots and Cox multivariate regression were computed using WinStat 2015 (R.Fitch Software, Staufen, Germany).

### Statistical Analysis

Statistical analysis was performed using Microsoft Excel software. Significant differences were analyzed using student’s *t* test and two-tailed distribution. Results were considered to be statistically significant if *p* < 0.05. Results were expressed as mean ± SE between triplicate experiments performed thrice.

## Additional Information

**How to cite this article**: Xie, B. *et al*. Benzyl Isothiocyanate potentiates p53 signaling and antitumor effects against breast cancer through activation of p53-LKB1 and p73-LKB1 axes. *Sci. Rep.* **7**, 40070; doi: 10.1038/srep40070 (2017).

**Publisher's note:** Springer Nature remains neutral with regard to jurisdictional claims in published maps and institutional affiliations.

## Supplementary Material

Supplementary Data
