# Supplementary material for: Benzyl Isothiocyanate potentiates p53 signaling and antitumor effects against breast cancer through activation of p53-LKB1 and p73-LKB1 axes
Source: Sci Rep. 2017 Jan 10;7:40070. doi: 10.1038/srep40070 (PMC5223184; doi:10.1038/srep40070)

Benzyl Isothiocyanate potentiates p53 signaling and antitumor effects against breast cancer through activation of p53-LKB1 and p73-LKB1 axes

Bei Xie[1§], Arumugam Nagalingam[1§], Panjamurthy Kuppusamy[2], Nethaji Muniraj[1], Peter Langford[1], Balázs Győrffy [3], Neeraj K. Saxena[2*] and Dipali Sharma[1*]

[1]Department of Oncology, Johns Hopkins University School of Medicine and the Sidney Kimmel Comprehensive Cancer Center at Johns Hopkins, Baltimore MD 21231, [2]Department of Medicine, University of Maryland School of Medicine, Baltimore MD 21201, [3] MTA TTK Momentum Cancer Biomarker Research Group, H-1117 Budapest, Semmelweis University, Dept. of Pediatrics, H-1094 Budapest, Hungary

Supplemental Material

Supplemental Methods

Cell culture and reagents Authentication of cell lines was done by analysis of known genetic markers or response (e.g., expression of estrogen receptor and p53 and estrogen responsiveness) and all cells were cultured for less than 3 months before reinitiating cultures and were routinely inspected microscopically for stable phenotype. Breast cancer cells were seeded at a density of 1 X 10^6 /100-mm tissue culture dishes for treatments and the culture media were changed to serum free media containing treatments as indicated after 16 hours of serum starvation. Benzyl Isothiocyanate (BITC) was procured from LKT Laboratories (St. Paul, MN, USA). Antibodies for p53, phospho-p53-ser15, phospho-p53-ser18, p21, BAX, Lamin B, phospho-ERK, ERK, phospho-PRAS40, PRAS40, MDM2, PUMA, cleaved PARP, PARP, DR5, LKB1 and p73 were purchased from Cell Signaling Technology (Danvers, MA). β-Actin antibody was purchased from Sigma-Aldrich (St. Louis, MO).

Clonogenicity assay To perform colony formation assay, breast cancer cells (single-cell suspension) were plated in 12-well plates at a density of 250 cells per well overnight. The following day, cells were treated with BITC and the medium was replaced with fresh medium containing treatments every 3 days. After a 10-day treatment period, the medium was removed and colonies were stained with crystal violet (0.1% in 20% methanol). Colony numbers were assessed visually and colonies containing >50 normal-appearing cells were counted. Pictures were taken using a digital camera.

Anchorage-independent growth assay Anchorage-independent growth of breast cancer cells was assayed by colony formation in soft agar. Briefly, equal volumes of agar (1.2%) and complete medium were mixed to make 0.6% agar growth medium solution in 6-well tissue culture plates. Cells ($2x10^3$ cells/well) were suspended in media with or without BITC treatment followed by mixing with equal volume of agarose (0.6%). Cell suspension-agarose mix (2 ml) was then added to each well. Plates were incubated at 37ºC with 5% CO_2 in a humidified incubator for 3 weeks, and media with or without BITC treatment were added every 3 days. Colonies were stained with 0.005% crystal violet in PBS for 1 hour at room temperature and observed using Olympus IX50 inverted microscope. Colonies were counted in five randomly selected fields at 10x magnification. Results are expressed as an average number of colonies counted per micro-field.

Cell viability assay Cell viability assay was performed by estimating the reduction of XTT (2, 3-bis(2-methoxy-4-nitro-5-sulfophenyl)-2H-tetrazolium-5-carboxyanilide), using a commercially available kit (Roche Applied Science, Indianapolis, IN) following manufacturer's instructions. Breast cancer cells were plated in 96 well plates at an initial density of 4×10^3 cells / well for 24 hours followed by BITC treatment as indicated. XTT labeling reagent was added to each culture well to attain a final concentration of 0.3mg/ml. After 4 hour exposure at 37^0C, absorbance was measured between 450 and 690nm using a 96 well plate reader (SPECTRAmax PLUS, Molecular Devices, CA). Pilot experiments verified that the cell densities used in experiments performed were within the linear range of the XTT assay. A standard curve was prepared using cell densities from 1×10^3 to 1×10^6, and the results were calculated with respect to the number of cells.

Mammosphere assays were performed as previously described [34] and spheres (>50 µm) were counted (24). Single cells were plated in ultra-low attachment plates (Corning) at a density 500 to 5,000 cells/well in serum-free mammary epithelium basal medium (Lonza) supplemented with 1% penicillin/streptomycin, B27 (1:50, Invitrogen-Life Technologies), 5 µg/mL insulin, 1 µg/mL hydrocortisone (Sigma), 20 ng/mL EGF (R&D Systems), 20 ng/mL basic fibroblast growth factor (Stem Cell), and 2-mercaptoethanol. BITC was added as indicated to the media. The mammospheres were counted under an inverted microscope.

Apoptosis analysis Cells (10^5 cells per well) were seeded in triplicate in 6-well plates and treated with BITC for 24 hours. Cells were then harvested and stained with Annexin-V and propidium iodide (PI) (BioVision, Mountain View, CA). The cells were washed with PBS and re-suspended in a binding buffer (HEPES buffered saline solution supplemented with 2.5 mM CaCl2), and then subjected to fluorescence-activated cell sorting (FACS) analysis by a flow cytometer (Becton Dickinson). Apoptotic cell death was determined by counting the cells that stained positive for Annexin-V.

Preparation of subcellular fractions* and *immunoblotting Cellular cytosolic and nuclear fractions were prepared by incubating cells in 100 µl of ice-cold lysis buffer [10mM Tris-Hcl (pH 7.4), 10mM NaCl, 3mM $MgCl_2$, 0.5% NP-40, 2mM DTT and 0.1mM PMSF]. The lysates were incubated for 5min on ice followed by centrifugation at 4,000g for 10min at 4^0C to precipitate nuclei. Supernatant was stored as cytoplasmic fraction. Nuclear pellet was incubated with 100 µl of ice-cold extraction buffer [20mM Tris-Hcl (pH 7.9), 0.42M KCl, 0.2mM EDTA, 10% Glycerol, 2mM DTT and 0.1mM PMSF] for 10min followed by centrifugation at 12,000g for 10min at 4^0C to clear the nuclear debris. Total protein was quantified using the Bradford protein assay kit (Biorad, Hercules, CA). Equal amount of protein was subjected to western blot analysis. Protein lysates were resolved on sodium-dodecyl sulfate polyacrylamide gel, transferred to nitrocellulose membrane, and western blot analysis was performed. Immunodetection was performed using enhanced chemiluminescence (ECL system, Amersham Pharmacia Biotech Inc., Arlington Heights, IL) according to manufacturer's instructions.

LKB1 stable knockdown using Lentiviral short hairpin RNA. Five pre-made lentiviral LKB1 short hairpin RNA (shRNA) constructs and a negative control construct created in the same vector system (pLKO.1) were purchased from Open Biosystems (Huntville, AL). Paired LKB1 stable knockdown cells were generated following our previously established protocol. Lentiviral helper plasmids (pCMV-dR8.2 dvpr and pCMV-VSV-G) were obtained from Addgene (Cambridge, MA). Transient lentivirus stocks were prepared following the manufacturer's protocol. One day before transfection, 1.5×10^6 293T cells were plated in 100-mm dishes. Cells were co-transfected with shRNA constructs (3µg) together with 3µg pCMV-dR8.2 dvpr and 0.3µg pCMV-VSV-G helper constructs. Two days later, viral stocks were harvested from the culture medium and filtered to remove

non-adherent 293T cells. To select for the MCF7 and MDA-MB-231 cells that were stably expressing shRNA constructs, cells were plated at subconfluent densities and infected with a cocktail of 1mL of virus-containing medium, 3mL of regular medium, and 8μg/mL polybrene. Selection with 0.5 to 2μg/mL of puromycin was started 48h after lentivirus infection. After four weeks of selection for MCF7 and MDA-MB-231 cells, monolayers of stably infected pooled clones were harvested for use and cryopreserved.

Phospho-Antibody Array Analysis The phospho-antibody array analysis was performed using the Proteome Profiler Human Phospho-Kinase Array Kit ARY003 from R&D Systems according to the manufacturer's instructions. Breast cancer cells were treated with BITC and lysed with Lysis Buffer 6 (R&D Systems). Preblocked nitrocellulose membranes of the Human Phospho-Kinase Arrays were incubated with 300 μg of cellular extracts overnight at 4 °C on a rocking platform, washed three times with 1x Wash Buffer (R&D Systems) followed by incubation with a mixture of biotinylated detection antibodies and streptavidin-HRP antibodies. Chemiluminescent detection reagents were used to detect spot densities. Array images were analyzed using the GeneTools image analysis software (Syngene). Every spot was subtracted by the averaged background level from negative control spots and normalized by the density levels of its own positive control spots. The averaged density of duplicated spots representing each phosphorylated kinase protein was determined and used for calculating the relative changes in phosphorylated kinase proteins. The list of target capture antibodies is available at http://www.rndsystems.com/pdf/ARY003.pdf.

Immunofluorescence and confocal imaging Breast cancer cells ($5x10^5$ cells/well) subjected to immunofluorescence analysis as described (23). Fixed and immunofluorescently stained cells were imaged using a Zeiss LSM510 Meta (Zeiss) laser scanning confocal system configured to a Zeiss Axioplan 2 upright microscope with a 63XO (NA 1.4) plan-apochromat objective. All experiments were performed multiple times using independent biological replicates.

Chromatin immunoprecipitation (ChIP) ChIP analyses were performed using our published procedure . Chromatin samples were sonicated on ice three times for 10 seconds each (i.e., until the average length of sheared genomic DNA was 1 to 1.5 kb) followed by centrifugation for 10 minutes. The immunoprecipitated

DNA was ethanol precipitated and re-suspended in 25 μl of water. Total input samples were re-suspended in 100 μl of water and diluted 1:100 before PCR analysis. Initially, PCR was performed with different numbers of cycles and/or dilutions of input DNA to determine the linear range of amplification; all results shown fall within this range. Following 28-30 cycles of amplification, PCR products were run on 1% agarose gel and analyzed by ethidium bromide staining.

TUNEL assay Breast tumor sections were deparaffinized, rehydrated and then used to visualize apoptotic bodies by TUNEL staining. TUNEL staining was conducted according to the supplier's instructions. Four to five randomly selected and nonoverlapping fields were imaged to score TUNEL-positive cells.

Gene chip database construction To validate the correlation between clinical outcome related to genes influenced by BITC, we performed a meta-analysis of publicly available datasets. For this, a gene expression database comprising microarray data with clinical annotation for breast cancer patients was setup up as described previously (30). As most of the genes were only present in the HGU133plus2 microarrays, only patient samples measured using this platform were included in the analysis and of these, only patients with relapse-free survival time were examined (n=1819). We matched genes between the BITC regulated gene list and gene chip identifiers using the HUGO gene nomenclature database (http://www.genenames.org). As several of the genes were detected by multiple probe sets in the gene expression analyses, we removed repeated entries so that each gene was included only once in the final analysis. In this, we selected the most robust probe set to represent a given gene using JetSet (https://cran.r-project.org/web/packages/jetset/index.html). Kaplan-Meier survival plots and Cox multivariate regression were computed using WinStat 2015 (R.Fitch Software, Staufen, Germany). To establish two cohorts, the lower quartile of the expression of the gene was used as cutoff. Multivariate analysis was performed using ER status, HER2 status and MKI67 expression as a surrogate marker for proliferation (31). Statistical significance was set at $p<0.05$.

Legends to Supplemental Figures

Supplemental Figure 1 BITC inhibits growth, clonogenicity, anchorage-independent growth, and mammosphere-formation, increases apoptosis of breast cancer cells. (A) Breast cancer cells were treated with various concentration of BITC and subjected to XTT assay**.** *, P<0.01, compared with controls. Vehicle-treated cells are denoted with the letter "C". **(B)** Clonogenicity of breast cancer cells treated with various concentrations of BITC (as indicated). **(C)** Soft-agar colony-formation of breast cancer cells treated with BITC for three weeks. Histogram represents average number of colonies counted (in six micro-fields). *, P<0.001, compared with controls. Vehicle-treated cells, denoted with the letter "C". **(D)** Breast cancer cells were treated with 2.5 µM BITC and subjected to mammosphere formation. Vehicle treated cells are denoted as (C). The graph shows the number of mammospheres. $^{*}p < 0.05$, compared with controls. **(E)** MCF7 and HBL-100 cells were treated with 2.5 µM BITC and subjected to Annexin V/PI staining. $^{*}p < 0.01$, compared with controls. **(F)** MCF7 and HBL100 cells were treated with 2.5 µM BITC, total protein lysates were subjected to immunoblot analysis using cleaved PARP, total PARP antibodies. Actin was used as a control. **(G)** MCF7 and HBL100 cells treated with 2.5 µM BITC were subjected to TUNEL assay. Bar diagram shows number of TUNEL-positive cells. *, P<0.001, compared with vehicle controls (C).

Supplemantal Figure 2 BITC-treated xenograft tumors exhibit reduced expression of anti-apoptotic proteins. (A, B) Tumors from vehicle (V) and BITC-treated mice were subjected to immunohistochemical (IHC) analysis using XIAP and Survivin antibodies. Bar diagrams show quantitation of IHC-analysis. Columns, mean (n =8); bar, SD. * significantly different (P< 0.005) compared with control.

Supplementary Figure 3 BITC does not alter p53 transcription. Total RNA isolated from MCF7 cells treated with 2.5 µM BITC for various time intervals as indicated was examined for p53 expression. β-Actin was used as control.

Supplemental Figure 4 ERK activation plays an important role in BITC-mediated growth-inhibition and apoptosis-induction. (A) MCF7 and HBL100 cells were transiently transfected with siERK-siRNAs for 48h and subjected to clonogenicity in the presence or absence of 2.5 μM BITC. Cells overexpressing ERK-CA are included as 'gain-of-function' controls. Histogram represents average number of colonies counted (in six micro-fields). *, P<0.01, compared with vehicle controls (C); **, P<0.01, compared with BITC-treated cells; ***, P<0.05, compared with BITC + ERKsi cells. **(B)** MCF7 and HBL100 cells were treated as in A and subjected to TUNEL assay. Bar diagram shows the number of TUNEL-positive cells. *, P<0.01, compared with vehicle controls (C); **, P<0.01, compared with BITC-treated cells; ***, P<0.01, compared with BITC + ERKsi cells.

Supplemental Figure 5 Overexpression of PRAS40 interferes with BITC-mediated growth inhibition. (A) MCF7 and HBL100 were transfected with constitutively-active PRAS40 (PRAS40-CA) and treated with 2.5 μM BITC as indicated followed by clonogenicity **(A)** and soft-agar colony assay **(B)**. *, P<0.001, compared with vehicle controls (C); **, P<0.005, compared with BITC-treated cells; #, P<0.005, compared with BITC + PRAS40-CA cells. **(C)** MCF7 cells were transiently transfected with p53-siRNA and control-si for 48h. Total RNA was isolated and subjected to real-time PCR analysis for the expression of p73. No significant change in p73 expression was observed.

Supplemental Figure 6 BITC inhibits mammosphere formation of MDA-MB-231 cells. (A) MDA-MB-231 cells were treated with 2.5 μM BITC and subjected to mammosphere formation. Vehicle treated cells are denoted as (C). The graph shows the number of mammospheres. $^{*}p < 0.05$, compared with untreated controls. **(B)** MDA-MB-231 cells were treated with 2.5 μM BITC, 0.05 μM RITA, 25 μM PRIMA1 alone or in combination as indicated and subjected to clonogenicity assay.

Supplementary Figure 7 Involvement of PUMA in BITC-mediated growth inhibition. (A) Total protein lysates from HCT116 PUMA+/+ and HCT116 PUMA-/- cells were examined for PUMA expression in an

immunoblot assay. **(B)** HCT116 PUMA+/+ and HCT116 PUMA-/- cells were treated with various concentrations of BITC as indicated and subjected to clonogenicity assay. **(C)** HCT116 PUMA+/+ and HCT116 PUMA-/- cells were treated with various concentrations of BITC as indicated and subjected to soft-agar colony formation assay. Bar-diagram shows number of soft-agar colonies. *, P<0.01, compared with vehicle controls (C).

Supplemental Figure 8 Involvement of BAX in BITC-mediated growth inhibition. (A) Total protein lysates from HCT116 BAX+/- and HCT116 BAX-/- cells were examined for BAX expression in an immunoblot assay. **(B)** HCT116 BAX+/- and HCT116 BAX-/- cells were treated with various concentrations of BITC as indicated and subjected to clonogenicity assay. **(C)** HCT116 BAX+/- and HCT116 BAX-/- cells were treated with various concentrations of BITC as indicated and subjected to soft-agar colony formation assay. Bar-diagram shows number of soft-agar colonies. *, P<0.01, compared with vehicle controls (C).

Supplementary Figure 9 BITC induces p73 recruitment on p21 promoter in MDA-MB-231 cells. (A) Soluble chromatin was prepared from MDA-MB-231 cells treated with 2.5 µM BITC as indicated and subjected to chromatin immunoprecipitation assay using p73 antibodies. IgG antibody was included as control. The purified DNA was analyzed by real-time quantitative PCR using primers spanning the p53-binding sites at p21 promoter and negative control primers. **(B)** Soluble chromatin was prepared from MDA-MB-231 cells treated with 2.5 µM BITC as indicated and subjected to chromatin immunoprecipitation assay using p73 and LKB1 antibodies. IgG antibody was included as control. The purified DNA was analyzed by real-time quantitative PCR using primers spanning the p53-binding sites at p21 promoter and negative control primers.

Supplementary Figure 1

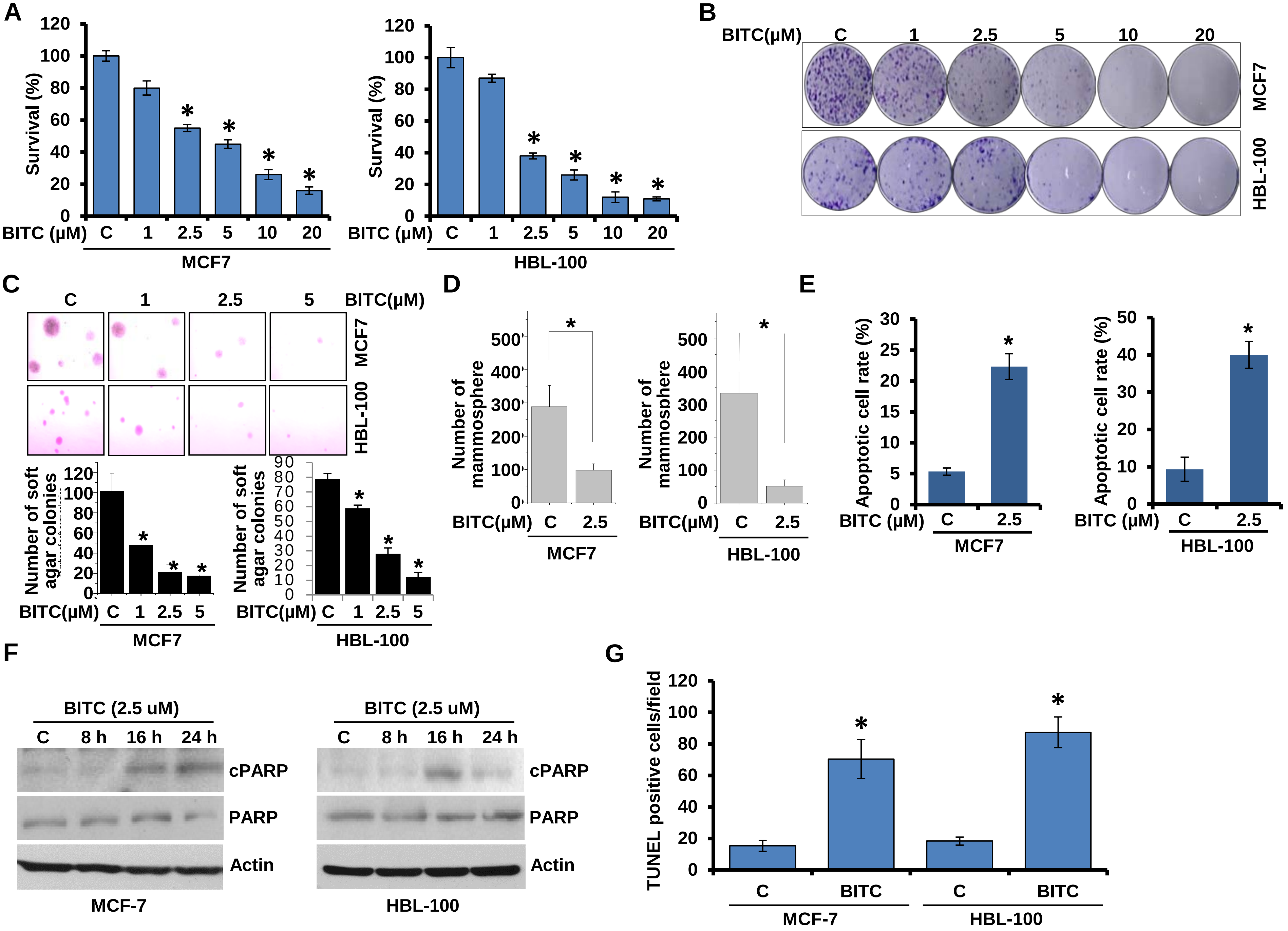

Supplementary Figure 2

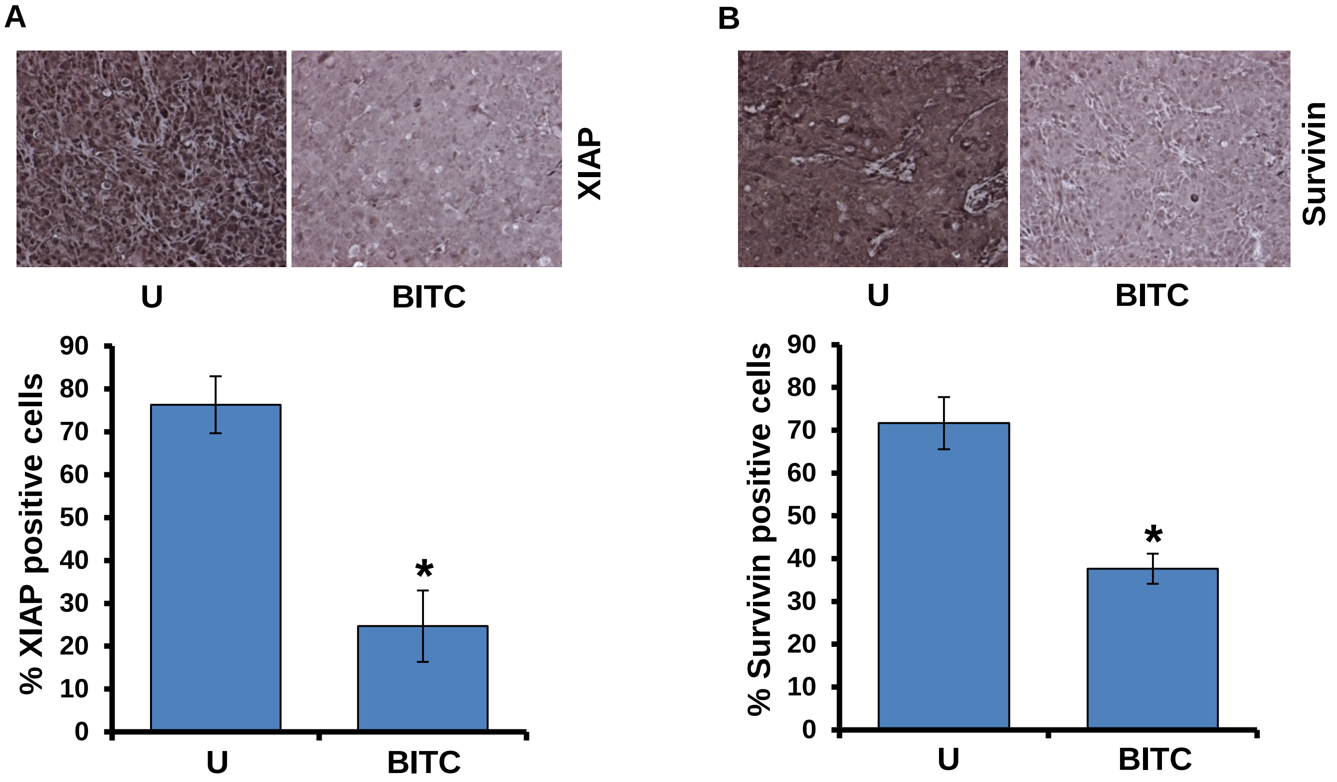

Supplementary Figure 3

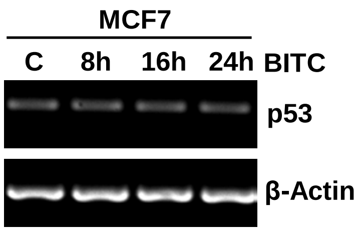

Supplementary Figure 4

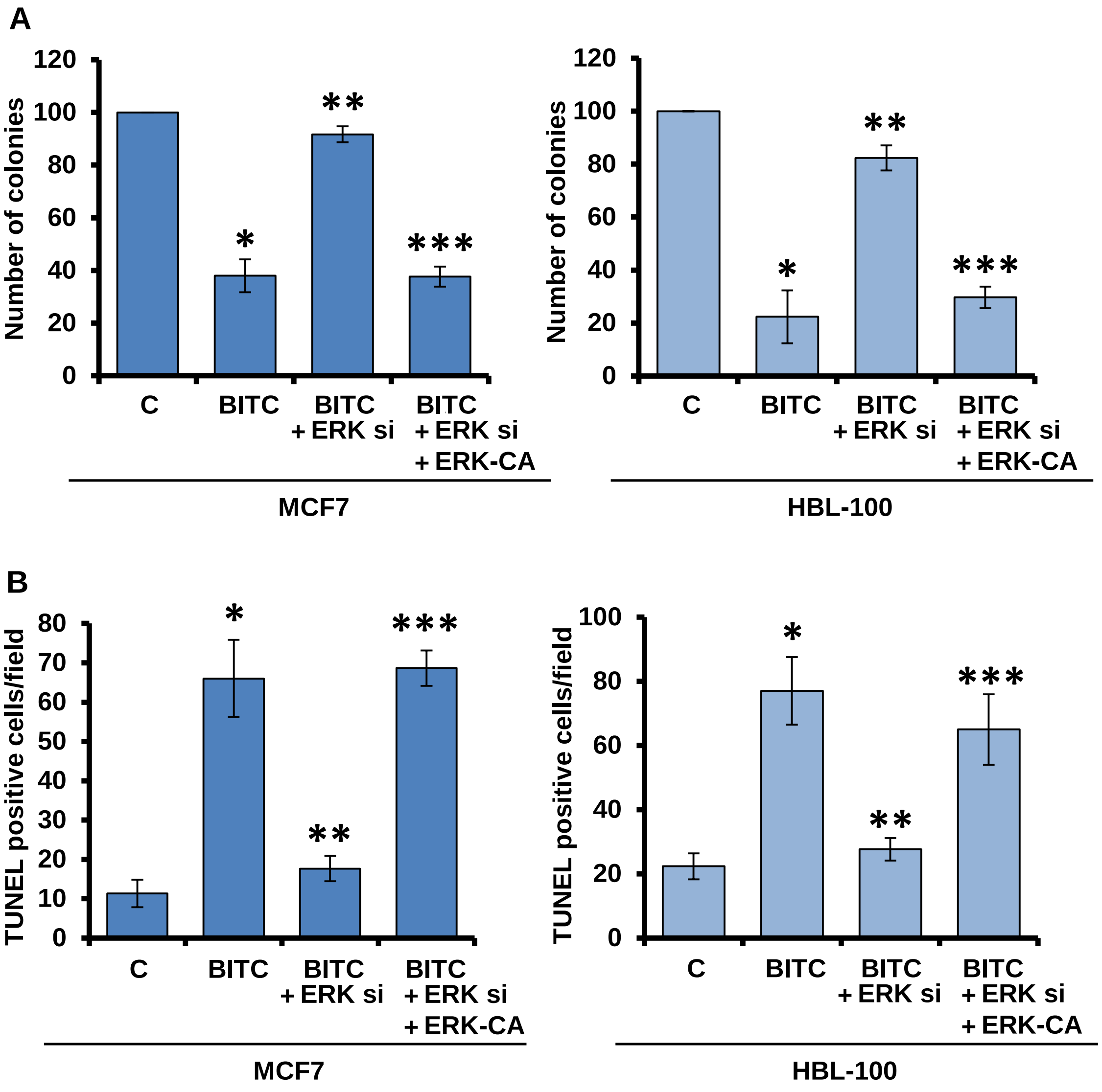

Supplementary Figure 5

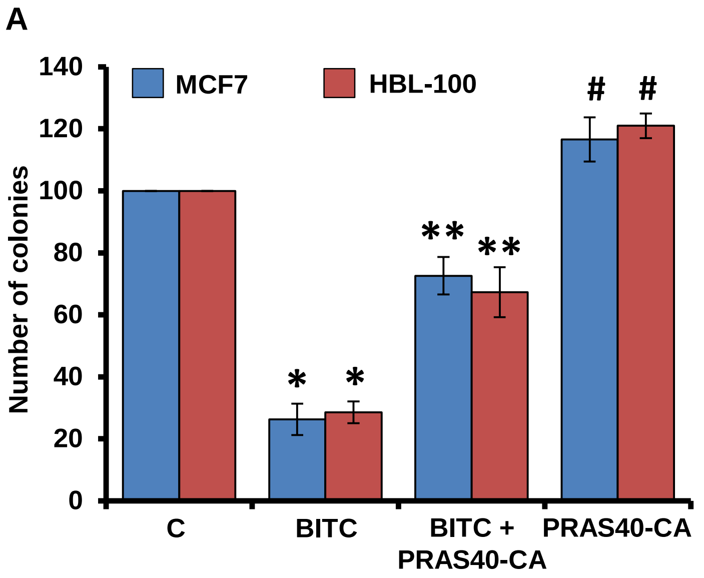

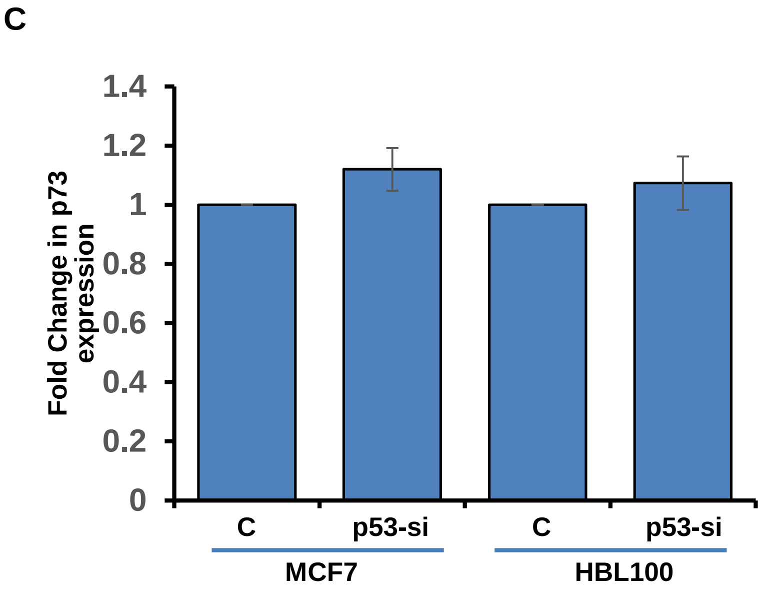

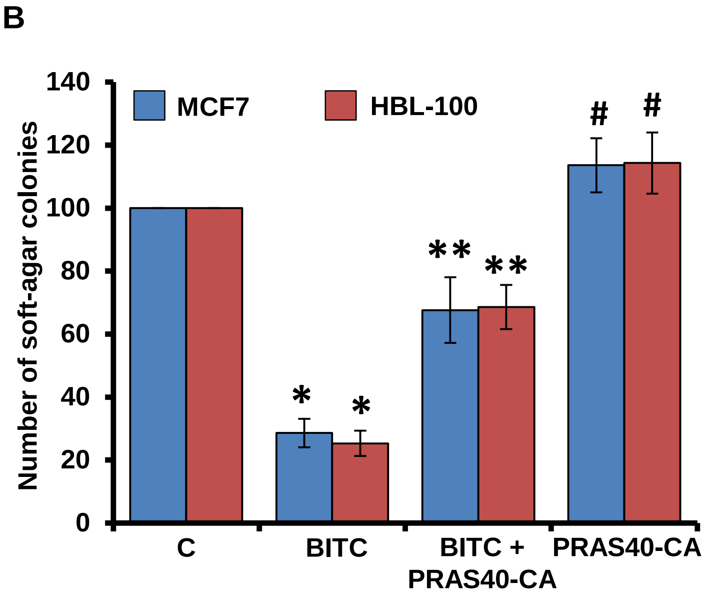

Supplementary Figure 6

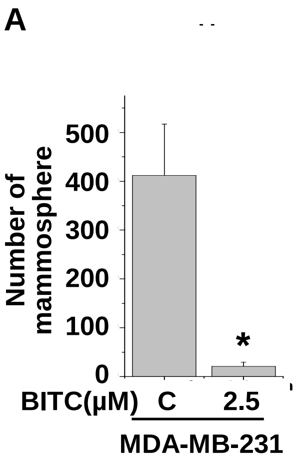

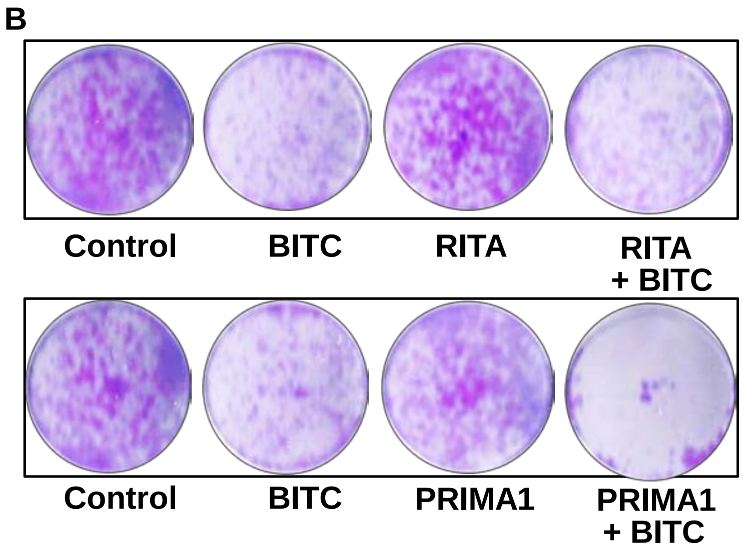

Supplementary Figure 7

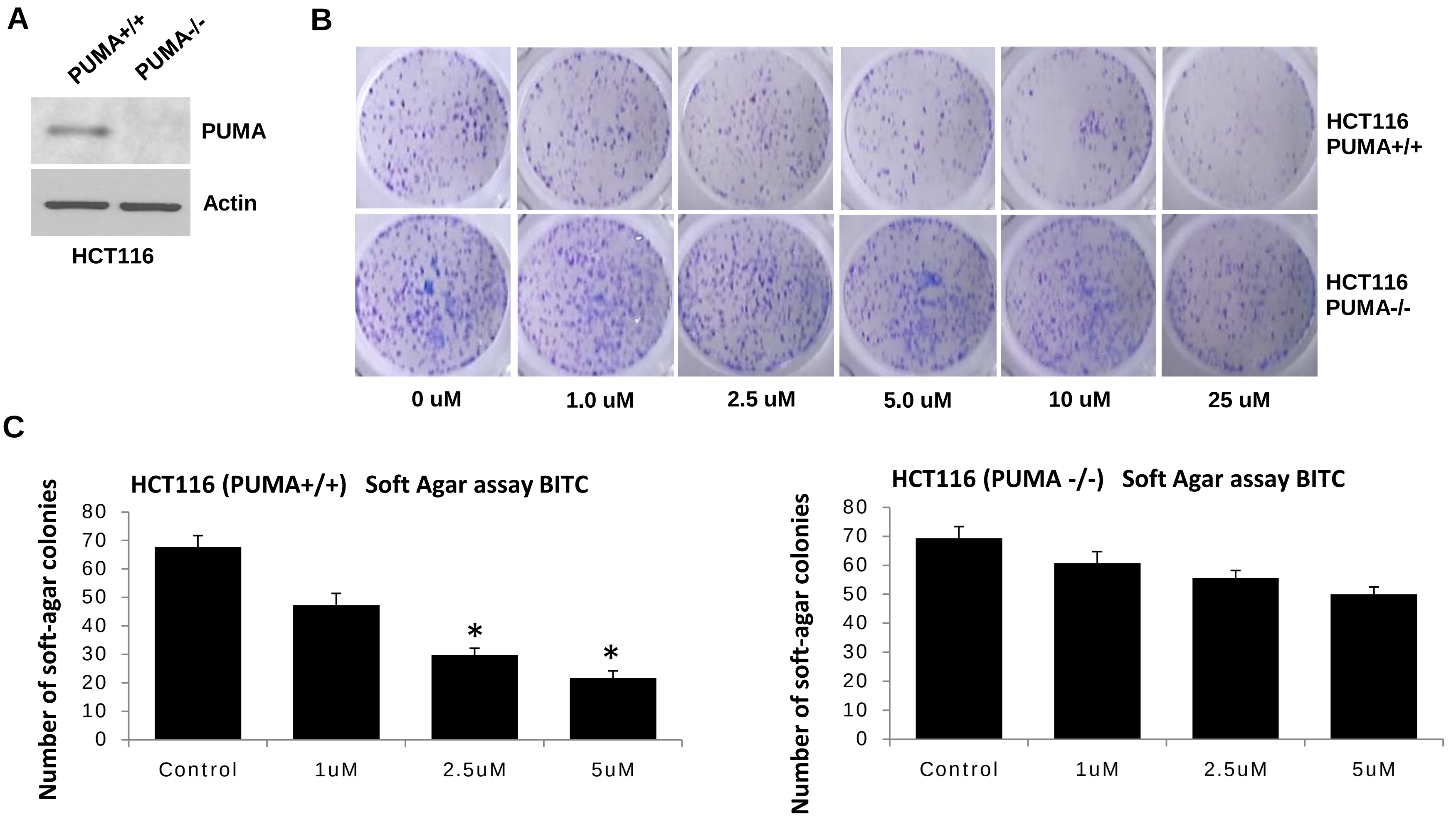

Supplementary Figure 8

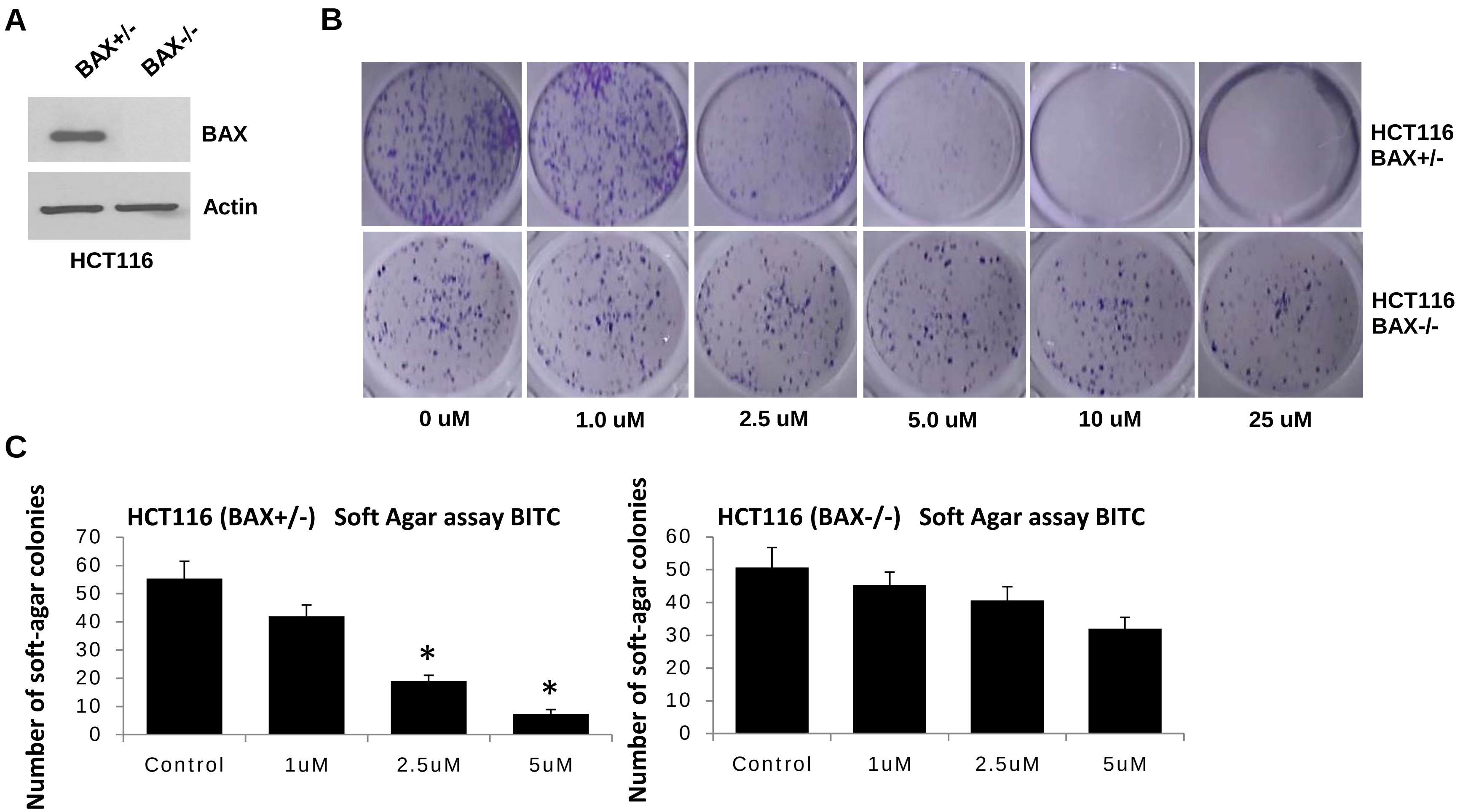

Supplementary Figure 9

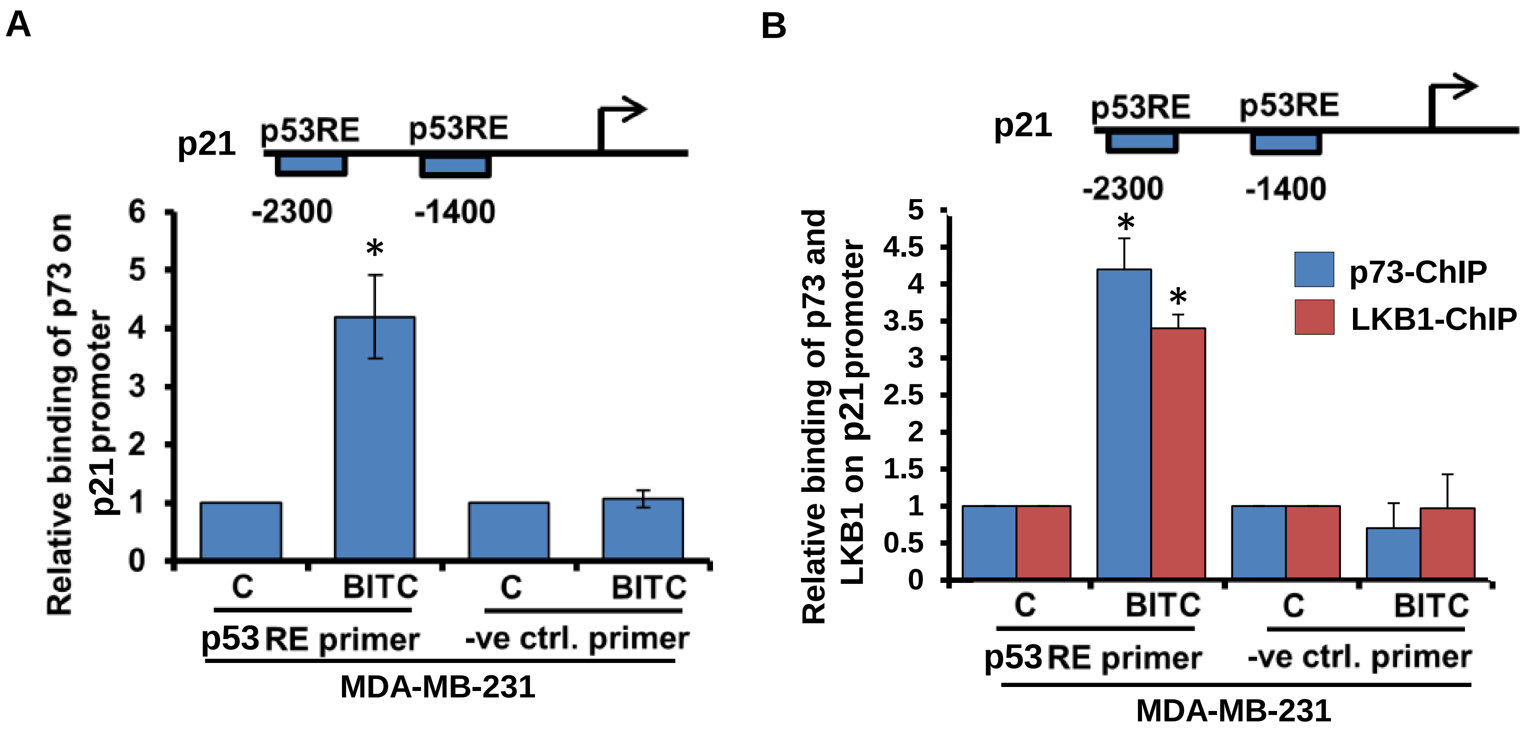

Supplement: Supplementary Data [file srep40070-s1.pdf]
